# Analytical Micromechanics Models for Elastoplastic Behavior of Long Fibrous Composites: A Critical Review and Comparative Study

**DOI:** 10.3390/ma11101919

**Published:** 2018-10-09

**Authors:** Yanchao Wang, ZhengMing Huang

**Affiliations:** School of Aerospace Engineering and Applied Mechanics, Tongji University, 1239 Siping Road, Shanghai 200092, China; 1008wangyanchao@tongji.edu.cn

**Keywords:** fibrous composites, analytical models, micromechanics, elastoplastic behavior

## Abstract

Elasto-plastic models for composites can be classified into three categories in terms of a length scale, i.e., macro scale, meso scale, and micro scale (micromechanics) models. In general, a so-called multi-scale model is a combination of those at various length scales with a micromechanics one as the foundation. In this paper, a critical review is made for the elastoplastic models at the micro scale, and a comparative study is carried out on most popular analytical micromechanics models for the elastoplastic behavior of long fibrous composites subjected to a static load, meaning that creep and dynamic response are not concerned. Each model has been developed essentially following three steps, i.e., an elastic homogenization, a rule to define the yielding of a constituent phase, and a linearization for the elastoplastic response. The comparison is made for all of the three aspects. Effects of other issues, such as the stress field fluctuation induced by a high contrast heterogeneity, the stress concentration factors in the matrix, and the different approaches to a plastic Eshelby tensor, are addressed as well. Correlation of the predictions by different models with available experimental data is shown.

## 1. Introduction

Fibrous composites have been vastly used in engineering due to their high specific moduli and strengths and admirable tailorability. Fibrous composites include long and short fiber reinforcements. Short fibrous composites are superior to the long ones in mass-production benefiting from their excellent formability. But the long fibrous composites have significant advantages over short ones on mechanical properties, based on the continuous long shape and improved alignment. In this work, the long fibrous composites are focused on. Unless specified, a composite in this research work is long fibrous.

The elastoplastic properties of a composite are important for the failure and strength evaluation [1,2], damage evolution [3], and dynamic damping analysis [4]. Mechanics of composites in an elastic range has been well-developed [5,6]. Based on an elastic homogenization and a linearization, an elastoplastic model for a composite can be established. However, much effort is still needed to improve the prediction accuracy and efficiency in a plastic range [7,8,9]. In general, elastoplastic theories for a composite can be classified into three categories by a length scale, i.e., macro scale, meso scale, and micro scale, i.e., micromechanics models.

**Figure 1:**
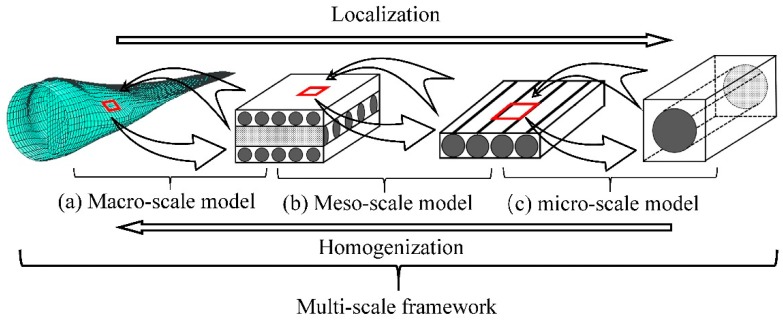
Schematic of a multi-scale framework: (**a**) macro-scale model; (**b**) meso-scale model; (**c**) micro-scale model.

As shown in Figure 1a, a macro model treats a laminate as a homogeneous anisotropic material so that the mechanical response of a composite structure such as a wind turbine blade can be analyzed. Generally, a macro model is incorporated into a finite element approach [10,11,12,13,14,15], in which elastoplastic properties of a laminate element can be obtained from experiments or a model at a smaller scale. For example, Dano et al. [16] developed a two dimensional numerical model for failure analysis of a fastened joint in a composite laminate, where the effective shear elastoplastic behavior of a laminate plate was described by a nonlinear internal variable obtained experimentally. Cooper and Warrior [17] conducted a finite element crash analysis for a composite structure, where an elastoplastic material element was implemented to describe the nonlinear behavior of the structure.

It should be noted that an anisotropic yield rule is necessary to describe the elastoplastic behavior, since a laminate is generally highly anisotropic. For example, Schmidt and Weichert [18] proposed an elastoplastic constitutive model for anisotropic shells using the Hill yield criterion [19]. Brünig [20] developed a numerical algorithm for anisotropic plates with the Tsai-Wu criterion to identify a yielding [21]. Under an assumption of an elastic-perfect plastic behavior, the Tsai-Hill yield criterion [22] was employed by Aykul et al. [23] in an elastoplastic analysis of a steel fiber reinforced aluminum matrix composite. With a macro model, analysis of complicated composite structures can be easily carried out by a numerical method. However, since composite materials are highly anisotropic and a plastic deformation is history dependent, the determination of the critical parameters in an anisotropic yield criterion is time and financial consuming [24,25]. Furthermore, how to establish a proper yield theory for a laminate with arbitrary lay-ups is still an open issue [26,27,28,29]. 

Meso scale models, also referred as layer-wise models, estimate mechanical properties of a laminate from the information of single layers [30,31,32,33,34,35]. In a meso scale approach (Figure 1b), a single layer is treated as a homogeneous and orthogonally anisotropic media. Its mechanical properties can be directly measured through experiments or obtained from a micromechanics model. One major problem for an elastoplastic model at a meso scale is how to describe the nonlinearity involved. Rotem [36,37] expressed the shear component in the stiffness tensor of a layer as a nonlinear function of a shear strain dependent parameter, while all the other components remained elastic. Pinho et al. [38] and Wolfe et al. [39] utilized a secant and a tangent linearization, respectively, to approximate the elastoplastic behavior of an individual layer. An instantaneous stiffness tensor was obtained by replacing the elastic modulus in it with a secant or tangent one.

To better understand the nonlinearity of a composite, it is necessary to consider an interaction between normal and shear stresses. Puck et al. [40] introduced a concept of stress exposure ratio to account for the interaction between the transverse normal and the in-plane shear stresses. Kress [41] expressed the transverse normal and the in-plane shear stresses in terms of the Hashin’s second and fourth invariants [42], respectively. Moreover, to fully address the stress interaction, anisotropic yield criteria for a layer are widely developed and employed [19,21,22,43,44,45,46,47,48,49,50]. For example, Sun et al. [51] proposed a one parameter plasticity model to describe the elastoplastic behavior of a UD (unidirectional) composite. The Tsai-Wu yield criterion was employed by Pisano et al. [45] to analyze the failure behavior of a pinned-joint composite laminate based on a layer-wise approach. However, it is still not easy to establish a general plasticity theory and a failure criterion for a single layer under a multiaxial load condition due to the high anisotropy. Besides, a meso scale model cannot capture detailed information at a constituent level, such as the field fluctuation in a matrix, the inclusion distribution and shape, and the imperfect interface between the constituents, which may be critical to a failure analysis.

A micro scale model evaluates the mechanical response of a composite from the microstructure and the properties of its constituent phases (Figure 1c). In an elastic range, numerical [52,53,54] and analytical micromechanics models [55,56] have been well developed. Making use of a linearization scheme, a micromechanics model in the elastic range can be extended to an elastoplastic range [9,57,58,59]. Compared with a meso or macro model, the yield condition at a micro scale is much easier to build. In addition, micromechanics models can significantly improve the design efficiency with composites, since only the constituent properties are required. A numerical micromechanics model can reflect the effect of complex microstructures on the mechanical responses of a composite [60,61,62,63,64]. However, it is very computational-consuming to do a full field microstructure-based analysis when a nonlinear behavior is involved [53,65,66,67]. The computational efficiency of an analytical model is significantly higher than that of a numerical one. Besides, analytical models can reveal physical mechanism of the mechanical response of a composite. But, it is difficult to establish an analytical model to describe the elastic-plastic behavior of a composite with all the complex microstructures considered. 

The complexity of a model determines whether it is convenient for engineering application. It is very difficult to compare the complexity from various aspects model by model. But it is practicable to give a distinct classification for models at different length scales by theoretical analysis. Regarding the plasticity, a macro-scale plasticity model for a composite depends on the fiber distribution density and lay-up information of a laminate. A meso-scale plasticity model is also sensitive to fiber configuration but regardless of the lay-up information. A micro-scale plasticity model is established for a homogeneous matrix when the fiber is seen as linear elastic. In such case, it only depends on information of the constituent matrix. Thus, it is reasonable to say that the complexity of plasticity decreases from macro-scale to micro-scale models. For the computation efficiency, in a macro-scale model, the input data of a laminate element are obtained from experiments. In a meso-scale model, such properties need to be calculated from a lamination theory. But in a micro-scale model, such information has to be calculated from information of fiber, matrix and their distribution. If all the calculation is analytical, the difference of computation is not obvious. But if the calculation is carried out by FEM, the difference in computational efficiency is significant. On the requirement of experiments, a macro-scale model needs to obtain input data from experiments of a laminate. When the fiber distribution or lay-up changes, experiments have to be re-conducted. But a micro-scale model only needs experiment data of constituent materials regardless of laminate geometry details. Thus, the experimental requirements decrease from macro-scale to micro-scale model. Lastly, for the complexity of modeling, a micro-scale model has to construct a model from fiber and matrix scale including fiber distribution and micro-cracks or voids. A macro-scale model can directly build a macro structure model by treating a laminate as a homogeneous material. Thus, the complexity of modeling increases from macro to micro-scale models in general. The complexity of models at different length scales are summarized in Table 1.

A practical way for the analysis of a composite structure is to take an approach of a multi-scale framework (MSF) [68,69,70,71,72,73,74]. As shown in Figure 1 in the MSF the models at different scales are built independently but are combined organically so that loads and material data can be transferred between the models. The micromechanics model plays a fundamental role in the MSF, because it provides bottom information. The establishment of a yield or failure criterion is more reliable at a micro scale [75,76,77,78,79,80,81]. Another kind of multi-scale model is so-called multiscale asymptotic homogenization method (MAHM) [82,83,84,85,86,87,88,89,90], in which a micromechanics model also plays a cornerstone role. It is generally applicable for a composite with periodic microstructures. In the MAHM, the fields of displacements, stresses, and strains are expressed in terms of small parameters which connect coordinates at different scales. Note that a homogenization function, the key to the MAHM, is addressed by a micromechanics model [70,91,92,93,94]. 

Furthermore, the plastic behavior and failure mechanisms of a composite can be well-understood only at the micro scale. Thus, it is necessary to make a comprehensive review on micromechanics models for elastic-plastic behaviors of composite materials. Chaboche et al. [95] presented a comparison between linearization schemes with and without an isotropic assumption on the matrix properties. It was pointed out that better results were obtained with the isotropic assumption [96]. Kanouté et al. [70] did a comprehensive review regarding multiscale homogenization models for nonlinear behaviors of composites. Effects of the linearization methods on the elastoplastic response were compared while the selection of an elastic homogenization was discussed. Charalambakis [59] provided a brief literature review on application of homogenization techniques to the prediction of, e.g., the elastic or inelastic properties, dynamic response and wave propagation of composite structures, without any comparative study. Klusemann and Svendsen [96] and Klusemann et al. [97] presented a comparison and benchmark study for multi-phase composites with elliptic and non-elliptic heterogeneities in an elastic range, respectively. Saeb et al. [98] and Matouš et al. [99] made reviews on multiscale homogenization methods for composites, focusing on numerical micromechanics. Ghossein and Lévesque [100] investigated prediction capability of various analytical micromechanics models by comparing with RVE (representative volume element) based FEAs (finite element analysis) in an elastic range. It was pointed out that the prediction accuracy was sensitive to the stiffness contrast of constituent materials [100].

Compared with a numerical micromechanics model, an analytical model has a distinct physical meaning which is essential to an in-depth understanding of the mechanical behavior of a composite. Besides, the computation efficiency of an analytical model is much more superior to a numerical one. In this work, a critical review and comparative study is made on most popular analytical elastoplastic micromechanics models for composites. Prediction deviations of the models induced by an elastic homogenization and linearization method are investigated. Some latest advancements are also accounted for in the comparison, including the stress concentrations in the matrix [101,102,103,104,105,106,107,108,109], the isotropization of an Eshelby tensor [65,110,111], an incremental-secant scheme [112,113,114,115], and Peng’s approach [116].

## 2. Review on Micromechanics Models

Micromechanics models are also referred to as homogenization models, meaning that the homogenized properties of a heterogeneous material, e.g., composite, are evaluated from properties of its constituent materials [59,117]. Micromechanics models can be separated into two classes, numerical and analytical [59,70]. In consideration of the advantage of computational efficiency, the analytical micromechanics models including empirical or semi-empirical ones are the focus of this work, whereas the numerical ones are briefly mentioned. Additionally, micromechanics models for a composite with an imperfect interface are also briefly reviewed.

### 2.1. Numerical Micromechanics Models

**Figure 2:**
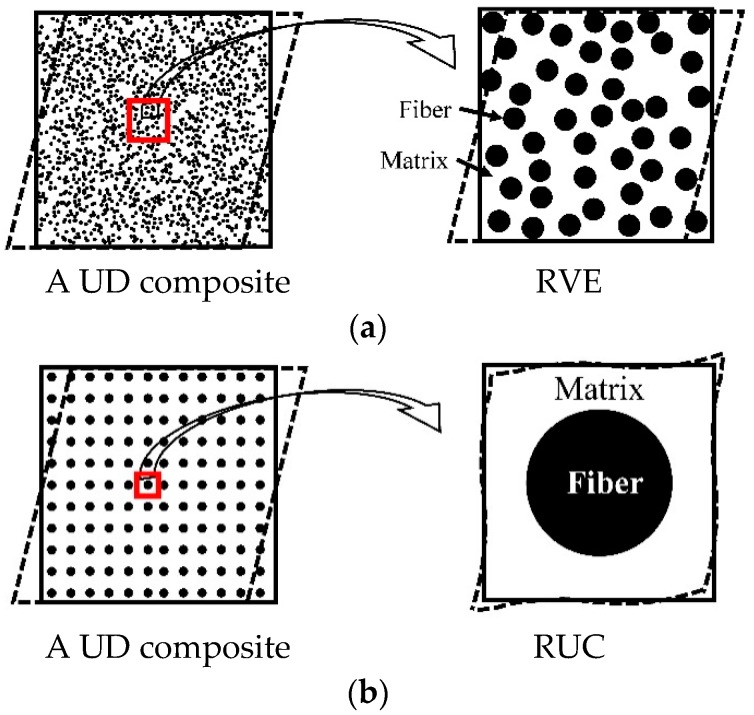
Schematic of a representative volume element RVE and a repeating unit cell (RUC) (solid line-undeformed, dash line-deformed). (**a**) An RVE for a unidirectional (UD) composite with randomly distributed fibers; (**b**) an RUC for a UD composite with periodically distributed fibers.

As shown in Figure 2, numerical models are based on either RVE (representative volume element) [118] or RUC (repeating unit cell) [54,58]. RVE based models apply to statistically homogeneous materials. Sufficient number of randomly distributed fibers was said to be necessarily contained in an RVE so that the microstructure of a composite could be reflected precisely [54,58]. Bohm et al. [119] generated an RVE containing 15 fibers using a randomly sequential adsorption algorithm to investigate the elastoplastic behavior of short fiber reinforced metal matrix composites. Kanit et al. [120] studied the relationship between the RVE size and its prediction capability for random composites. They pointed out that a larger RVE size gave better prediction accuracy but resulted in lower computational efficiency [120]. Heinrich et al. [121] claimed that an RVE containing at least 25 fibers could provide a satisfactory prediction accuracy. Determination of an RVE size has also been discussed by some other researchers [122,123,124,125].

RUC based models are applicable to a composite with periodic microstructures. Only one or several fibers are included in an RUC. The boundary condition, a critical point of an RUC model, has to be defined carefully to represent effect of different fiber distribution patterns and loads applied [54,58,89,126,127,128,129]. In some cases, the uniform boundary conditions are applied to RUCs. For example, Brockenbrough, et al. [130] investigated the effect of fiber distribution patterns on the response of a metal matrix composite with a uniform strain boundary condition. Also, similar treatment can be found in the work of Aboudi [131], Aghdam et al. [132], and Würkner et al. [133]. However, it was reported by Suquet [89] that an RUC model with such a uniform boundary condition could only give an upper or lower bound of the effective properties of a composite. Specifically, the effective stiffness of a composite would be overestimated by an RUC model with a uniform strain while underestimated with a uniform stress assumption. Suquet [89] gave a rigorous definition of the periodic boundary condition with which a better estimation of the effective properties was achieved. Xia, et al. [134,135] proposed a unified periodic boundary condition for an RUC under any combined loads. They indicated that the uniform boundary condition not only over constrained the RUC but might violate the boundary traction periodicity as well. A periodic boundary condition has been widely applied with both RVE [123,136,137,138] and RUC [139,140,141,142,143] models.

Elastic RVE and RUC models can be extended to nonlinear cases, providing that the nonlinear constitutive laws for the constituents are available. For example, based on a three-dimensional RVE model, Yuan and Lu [144] conducted a numerical investigation on the elastoplastic behavior of carbon nanotubes (CNTs) reinforced polymer composites. Hoang et al. [124] studied the effect of an RVE size on the prediction for the elastoplastic and elasto-viscoplastic behavior of a two-phase composite. Choosing an RUC, Aghdam et al. [132] analyzed the yield and collapse behavior of a metal matrix composite. With an RUC based multiscale model, Wan et al. [145] studied the compressive behavior of a braided composite after an impact accounting for the elastoplastic deformation of the matrix.

The computational quantity of a numerical micromechanics model is generally acceptable when dealing with a linear elastic problem. However, when a constituent e.g., matrix material becomes nonlinear, such as an elastoplastic or visco-elastoplastic behavior occurs, a common FEA approach is much more computationally-consuming [65,66,67,146]. To tackle this issue, a number of numerical micromechanics models with reduced computational effort have been developed. Examples include the Voronoi Cell Finite Element Method (VCFEM) [147,148,149], the Generalized Method of Cells (GMC) [131,150,151,152], the Finite Volume Direct Averaging Micromechanics (FVDAM) [153,154,155,156,157,158,159,160,161], and the Variational Asymptotic Method for Unit Cell Homogenization (VAMUCH) [86,162,163,164,165,166]. Reviews on them for nonlinear analysis of a composite can be found in Kanouté et al. [70] and Saeb et al. [98], among others.

### 2.2. Analytical Micromechanics Models

Analytical micromechanics models have been well developed for elastic problems but are still in progress for elastoplastic ones. Linearization is a practical way to establish an elastoplastic micromechanics model, in which a nonlinear response is discretized and approximated as a series of linear problems. With such a linearization, a micromechanics model can be extended to the analysis of an elastoplastic problem.

The selection of a proper micromechanics model is fundamental. The equivalent inclusion method pioneered by Eshelby [167] can be used to solve an eigenstrain problem with a single inclusion embedded in an unbounded matrix. As long as a fibrous composite is concerned, the interaction between the inclusion and the surrounding fibers is ignored in the Eshelby’s method. A self-consistent model (SCM) [55] is advanced from the Eshelby’s method by replacing the matrix phase with a medium whose properties equal to the effective properties of the composite. Although better in a prediction accuracy, the implicit formulae resulted from the SCM make it inconvenient for use in engineering. Moreover, the SCM may give a physically nonsense result when the inclusion is rigid or a void [168]. Afterwards, Mori and Tanaka developed a method [56] by keeping the same configuration of the Eshelby model but assigning the homogenized strain field of the matrix as that of the composite. Further, a generalized self-consistent method (GSCM) was proposed [169,170,171], dealing with a configuration that a single fiber surrounded by the matrix is embedded in an infinite homogeneous medium whose properties are the same as those of the composite. In addition, two or three phase concentric cylinder assembly (CCA) models are also widely used to determine the effective properties of the composite [170,172,173,174,175,176,177,178]. Note that the two-phase and three-phase CCA models are equivalent to the Mori–Tanaka and the GSCM, respectively, when the inclusions are cylindrical [168,179]. Moreover, a double inclusion approach [180,181,182] is proposed to evaluate the mechanical properties of a multiphase composite, of which the Mori–Tanaka model, the SCM, and the GSCM can be considered as a special case.

In addition to the theory of elasticity based models mentioned above, some semi-empirical analytical models are also well-known. With the iso-stress and iso-strain assumptions, the classical rule of mixture [183,184] is resulted. The rule of mixture can give good prediction for the longitudinal modulus and the major Poisson’s ratio but deliver poor estimation for the other elastic properties of a composite. Based on experimental results, Chamis et al. [185,186] achieved a model called Chamis’ model, which can be considered as a modified version of the rule of mixture. Much better prediction in the transverse and shear moduli is generally seen. Halpin and Tsai [187] presented a simplified version of the SCM, known as Halpin–Tsai’s equations.

Huang [188] developed a unified elasto-plastic bridging model in which a bridging tensor is employed to link the homogenized stresses of the matrix with those of the fiber. The bridging tensor elements can be classified as dependent and independent. Whereas the dependent elements are determined from the symmetric condition of the overall compliance tensor of the composite, the independent ones are expressed in the form of Taylor series expansions with respect to the fiber and matrix property parameters. The expansion coefficients, independent of any constituent property, were determined based on existing micromechanics theories in an elastic range. It can be seen that the independent bridging tensor elements thus obtained are simplifications and modifications to the corresponding counterparts of the Mori–Tanaka model [179,189]. As long as a plastic deformation of a constituent e.g., matrix material is concerned, only the corresponding matrix property parameters in the Taylor expansion need to adjust. It has been shown that the bridging model can give better correlation between the predicted and measured effective elastic properties of UD composites than many other famous models [190,191,192,193].

The second step in the establishment of an elastoplastic model is the selection of a linearization. Hill [194] proposed an incremental linearization in which stress and strain increments were linked with an instantaneous stiffness or compliance tensor. Consider a homogeneous material subjected to uniaxial tension. An instantaneous Young’s modulus is defined as a tangent to the stress-strain curve of the material at the current stress analysis point. Hence, an incremental linearization scheme is also referred to as a tangent approach. The incremental linearization has been widely used due to its capability on history dependent cases [9,195,196,197]. Following Hill’s work [194], Berveiller and Zaoui [198] and Tandon and Weng [199] proposed a deformation linearization in which the total stress and strain were connected by a secant stiffness or compliance tensor. Under a uniaxial tension, a secant Young’s modulus is defined as the secant slope between an objective point and the initial point on the stress-strain curve. Puck and Schurmann [200] pointed out that the secant method offers the advantage of self-correction, meaning that the error induced by the secant method in the current load step will not be transferred to the next step whereas a tangent approach will be done due to its incremental nature. The deformation linearization, referred to as a secant approach, also gains a popularity in application benefiting from its simple formulation and good prediction accuracy [201,202,203]. In comparison, it is seen that the tangent model applied to non-proportional and non-monotonic loads whereas a secant model is generally restricted to proportional and monotonic cases [114,199,202,204,205,206,207]. In addition to these two linearizations, Dvorak [208] proposed a transformation field analysis (TFA) method. In the TFA, properties of the constituents keep elastic. The plastic strain in a constituent is taken as an eigenstrain whose effect on a composite response is reflected by an influencing function. The TFA has been implemented into an FEA for nonlinear analysis of composites [209].

It has been reported that the nonlinear stress strain curve evaluated by a tangent approach, a secant approach, or the TFA may be too stiff compared with experiments [95,210,211,212,213]. Existing attempts to address the too stiff problem are mainly in three kinds, i.e., improvements on a linearization, corrections on an equivalent stress, and modifications on the calculation of a plastic Eshelby’s tensor.

Regarding the first kind attempt, Molinari [214] suggested that the too stiff response might be amended by a full consideration of an interaction between plastic deformation of the fiber and matrix. He then proposed a non-incremental tangent linearization approach, which was validated by Molinari et al. [215] and Mecier et al. [216]. Using the same linearization [217], Masson et al. [218] and Zaoui et al. [219] proposed an affine formulation for an elastoplastic response of a composite. The affine formulation was further developed and validated by Pierard and Doghri [110], Pierard et al. [220], and Doghri et al. [221]. On the other hand, Wu et al. [112,113,114] believed that better results could be obtained by an incremental-secant scheme they proposed. In their approach, when the load was increased from the current to the next step, the effective properties of a composite were approximately estimated by the secant method. Afterwards, a fictitious elastic unload was introduced, from which residual stresses and strains induced by the plastic deformation were obtained and accumulated [112,113,114]. One feature of this incremental-secant scheme is in its applicability to non-proportional and non-monotonic load problems.

As for the second kind attempt, it was pointed out that the too stiff response might be resulted from an ignorance of a stress field fluctuation in the constituents [205]. Instead of calculating the von-Mises equivalent stress from homogenized stresses of the constituent materials in a composite, Qiu and Weng [205] and Hu [222] derived the von-Mises equivalent stress from an energy approach which can reflect the effect of stress fluctuation on yield behavior. Inspired by their work, Suquet [223] proposed a modified secant model, where the second-moment estimation for stresses was used to capture the stress fluctuation in the constituent phases of a composite. Independently, basing on the work of Talbot and Willis [224], Ponte Castaneda [225,226] proposed a rigorous variational principle to give bounds or estimates for nonlinear composites. It is interesting to find that the modified secant model by Suquet [223] coincides with the variational estimation by Ponte Castaneda [225,226,227] when the complementary energy of the constituent phases is a quadratic form of the stress tensor [228].

Regarding the third attempt, there are four approaches in the literature to determine a plastic Eshelby’s tensor. The first one [229,230,231,232] is named as anisotropic Eshelby tensor method, meaning that an instantaneous stiffness tensor of the matrix under an elastoplastic deformation is computed rigorously from a plasticity theory, such as Prandtl-Reuss model, and the corresponding Eshelby tensor is also anisotropic and evaluated through a numerical integration. This approach gives the most rigorous tensor but may result in a too stiff response [65,197,233,234]. The second is referred as isotropicalized matrix method. In this method, the instantaneous stiffness of a matrix can be approximated as isotropic, and the corresponding Eshelby tensor is isotropic as well [65,145,160]. Namely, the Eshelby tensor in a plastic region is similar to that in an elastic one. However, an isotropization has been demonstrated successful only for a proportional load case [212]. The third approach is designated as isotropicalized Eshelby tensor method. In this approach, an instantaneous stiffness tensor of the matrix remains anisotropic whereas the Eshelby tensor is determined according to an isotropic condition. Namely, only the elastic property of the matrix in the Eshelby tensor of an elastic region is replaced with a plastic counterpart of the matrix. This method was first proposed by Doghri and Ouaar [65], and was further applied by many other researchers [111,197,234,235,236,237,238,239]. However, Huang et al. [240] and Peng et al. [116] pointed out that the isotropization of the Eshelby tensor has no sound physical background. The fourth approach has been recently presented by Peng et al. [116]. At each load step, a reference elastic medium (REM) is introduced whose configuration and properties are identical to the instantaneous ones of the elastoplastic medium (EPM). Based on the REM, Peng et al. obtained a scheme to determine the Eshelby tensor with modified prediction on the elastoplastic behavior of a composite.

Very recently, Huang et al. [103,104,105,107,108,109,241] have shown that the homogenized stresses of the matrix should be converted into true values before an effective property of the composite can be determined in terms of the monolithic fiber and matrix properties. The conversion is achieved by multiplying the homogenized stresses with the respective stress concentration factors (SCFs) of the matrix due to introduction of the fiber, and all of the SCFs in relation to different loads under perfect as well as debonded fiber-matrix interface have been derived [103,104,105,107,108,109,241]. The elastic properties of the composite are independent of the stress values in the constituents, and thus the true stress concept plays no role. However, when the matrix is in a plastic deformation, the true stresses may result in an instantaneous stiffness of the matrix lowered down significantly in comparison with the homogenized counterparts. This is because essentially all of the SCFs are greater, and some are significantly higher, than 1 especially after the interface debonding. In turn, the composite stiffer response resulted from the homogenized stresses in the fiber and matrix can be satisfactorily addressed. 

**Figure 3:**
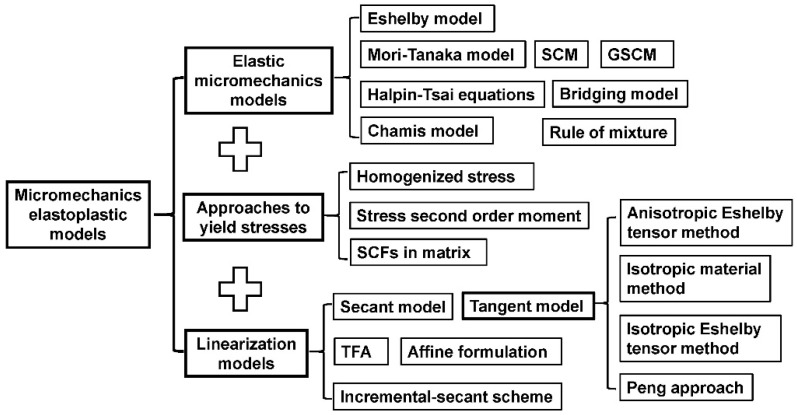
Establishment of elastoplastic models for composites.

To better understanding the establishment of an elastoplastic model, Figure 3 summarized the process of extending an elastic model to an elastoplastic range.

### 2.3. Micromechanics Models with Imperfect Interface

**Figure 4:**
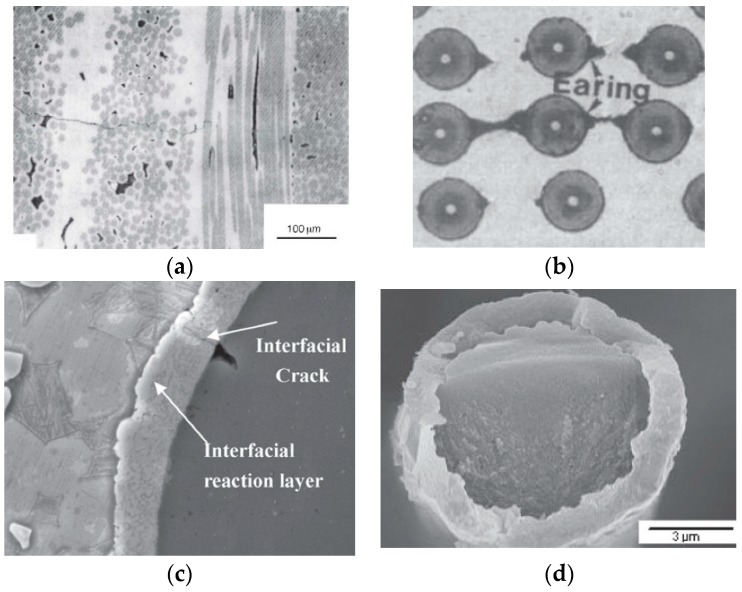
Imperfect interface phenomenon in fibrous composites. (**a**) Crack growth in a SiC/SiC woven composite under cyclic load [242]; (**b**) Ear-hole formation in a SiC/Ti-6Al-4V composite [243]; (**c**) Interphase produced by chemical reaction in a SiC/Ti-6Al-4V composite [244]; (**d**) BN coated T300 fiber [245].

The afore-mentioned models/theories are mainly built for a composite with a perfect interface. A perfect interface condition means that the stress and displacement fields are continuous at the interface in-between the fiber and matrix. Contrarily, imperfect interface condition implies that the stress or displacement field is discontinuous at the interface. Perfect interface condition is applicable for most engineering applications of composite materials. However, imperfect interface situations do exist in some cases. For example, interface debonding occurs when a composite subjected to a fatigue load that exceeds its elastic limit, e.g., Figure 4a [242]. Cracks or micro-voids are often observed at the interface in a thermo-pressure lamination process of a kind of metal matrix fibrous composite, e.g., Figure 4b [243]. In addition, an interphase is produced due to the chemical reaction between the fiber and matrix (see Figure 4c) [244]. Moreover, coating, also a kind of interphase, is often added to a fiber for the purpose of function design, as shown in Figure 4d [245]. Both the interface debonding/crack and the interphase can be classed to imperfect interface condition. It is reported that the effect of imperfect interface on mechanical properties of a composite is unignorable in some cases [246,247,248].

In elastic range, micromechanics models with an imperfect interface can be classified into two categories [249], i.e., interface model and interphase model. In an interface model, a crack-like zero-thickness interface is employed to characterize the imperfect interface between fiber and matrix. The stress or displacement fields across the interface are discontinuous. Obviously, an interface model is proper for the cases shown in Figure 4a,b. The linear-spring model [250,251] and interface stress model [252,253] are the two well-known interface models. At the interface, the former assumes that the stress field is continuous while the displacement field is not. The displacement variance is proportional to the stress. The latter assumes a continuity of displacement but discontinuity of stress. This model is generally used to describe interface compression phenomenon. Other models, like dislocation-like model [254], interface sliding model [255,256], and the anti-interpenetration model [257] can be seen as extensions of the linear-spring and interface stress models. An interphase model adds a layer between the fiber and matrix in a two-phase concentric cylindrical assembly (CCA) [174]. The mechanical property of the layer is different from those of the fiber and matrix. Clearly, an interphase model is appropriate for the cases shown in Figure 4c,d. Hashin [174] gave an exact solution for a three-phase CCA model with thin interphase. Further, Benveniste [178] extended Hashin’s results to a three-phase CCA model with thick interphase. It is pointed out that an interphase model is equivalent to an interface model when the interphase is thin and soft [249,258,259]. The exact solution of an interphase model is complex. Based on a two-phase bridging model and an equivalent fiber method, Wang [179,189,260] proposed a simplified analytical three-phase model for the analysis of composites with imperfect interface.

In nonlinear range, for a composite with an imperfect interface, it is reasonable to assume that the nonlinearity of a composite mainly comes from the elasto-plastic behavior of the matrix and the progress damage of the interface. Chang et al. [261] developed a progressive damage model for a composite laminate. But their model is in macro-scale, meaning that the stress analysis at micro scale is not available. In addition, the contribution of matrix and interface nonlinearity cannot be distinguished. Ju et al. [262] proposed a micromechanics interface damage model. In their work, the interface damage was approximately described by an inhomogeneity with transverse isotropy. However, their work is only valid for particle reinforced composites. The cohesive element method [3,263] has been widely used in simulation of a composite with an imperfect interface. The cohesive element method has sound physical background and is powerful in the analysis of interface crack propagation. However, the cohesive model has to be implemented into a micro-scale FEM, thereby being of high cost in computation resource. For engineering applications, it is desirable to develop a model with abilities in micro-scale damage analysis, satisfied prediction accuracy, and high computational efficiency.

It is noted that models with imperfect interface have been vastly investigated. But, for most engineering applications, the perfect interface assumption is good enough for mechanical analysis. In this work, a composite with perfect interface is mainly focused on.

## 3. Comparison on Elastic Theories

The selection of a proper elastic micromechanics theory is in the fundamental step to establish an elastoplastic model. In this section, summary of different elastic micromechanics models is made. Then, the elastic models are evaluated regarding their capabilities in predicting elastic properties of UD composites. Further, based on a tangent linearization, the elastic models are extended to be valid in an elastoplastic range. A quantitative comparison for them is shown in the next section.

### 3.1. General Framework

**Figure 5:**
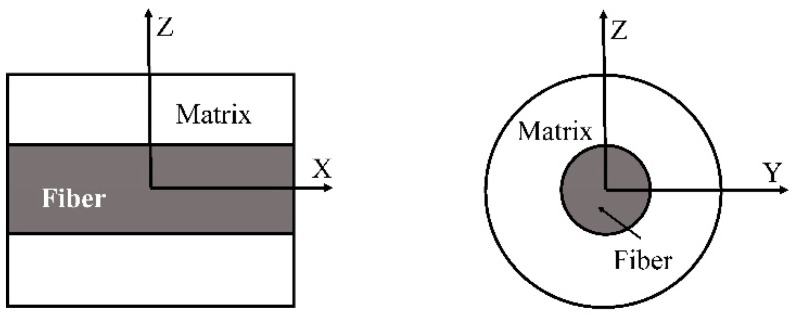
Schematic of an RUC for a UD composite.

Consider an RUC shown in Figure 5. The stress and strain components must be homogenized with respect to the volume of the RUC through Equations (1) and (2).

(1) $$\;\bar{\sigma }=\frac{1}{V}\int \sigma \left(x\right)dV\;$$

(2) $$\;\bar{\varepsilon }=\frac{1}{V}\int \varepsilon \left(x\right)dV\;$$

where σ(x) and ε(x) are, respectively, point-wise stress and strain tensors, $\bar{\sigma }$ and $\bar{\varepsilon }$ the homogenized counterparts. Since only the homogenized quantities are dealt with, the over bars are omitted.

For a two-phase composite with fiber and matrix constituents, the stress and strain of a composite are given by Equations (3) and (4).

(3) $$\;\sigma =V_{f}\sigma_{f}+V_{m}\sigma_{m}\;$$

(4) $$\;\varepsilon =V_{f}\varepsilon_{f}+V_{m}\varepsilon_{m}\;$$

*f* and *m* designate the fiber and matrix, respectively. A quantity with no suffix belongs to a composite. Following Hill [264], there are two fourth-order strain and stress concentration tensors, $A_{r}$ and $B_{r}$, as shown in Equations (5) and (6).

(5) $$\;\varepsilon_{r}=A_{r}:\varepsilon ,\;r=f,m\;$$

(6) $$\;\sigma_{r}=B_{r}:\sigma ,\;r=f,m\;$$

Let M and L denote compliance and stiffness tensors with Equations (7) and (8).

(7) ε=M:σ, σ=L:ε,

(8) $$\varepsilon_{r}=M_{r}:\sigma_{r},\;\sigma_{r}=L_{r}:\varepsilon_{r},\;r=f,m\;$$

Then, the effective stiffness and compliance tensor are given by Equations (9) and (10).

(9) $$\;L=L_{m}+V_{f}\left(L_{f}-L_{m}\right):A_{f}\;$$

(10) $$\;M=M_{m}+V_{f}\left(M_{f}-M_{m}\right):B_{f}\;$$

Equation (11) is also useful:

(11) $$B_{r}=L_{r}:A_{r}:M,\;r=f,\;m$$

The strain (stress) concentration tensor, $A_{r}$ ($B_{r})$, that connects a strain (stress) tensor of a constituent with that of a composite is named as a global strain (stress) concentration tensor. Very often, it is easier to establish a model with a local concentration tensor. Let $T_{f}$ and $P_{f}$ represent the local strain and stress concentration tensor, respectively, such as Equations (12) and (13).

(12) $$\;\varepsilon_{f}=T_{f}:\varepsilon_{m}\;$$

(13) $$\;\sigma_{f}=P_{f}:\sigma_{m}\;$$

The connections between the local and the global concentration tensors are found as shown in Equations (14) and (15).

(14) $$\;A_{f}=T_{f}:{\left(V_{m}I+V_{f}T_{f}\right)}^{-1}\;$$

(15) $$\;B_{f}=P_{f}:{\left(V_{m}I+V_{f}P_{f}\right)}^{-1}\;$$

Further, once the stiffness and compliance tensors, L and M, are known, the global concentration tensors are given (see Equations (16) and (17)).

(16) $$\;A_{f}=\frac{L-L_{m}}{V_{f}\left(L_{f}-L_{m}\right)}\;$$

(17) $$\;B_{f}=\frac{M-M_{m}}{V_{f}\left(M_{f}-M_{m}\right)}\;$$

**Figure 6:**
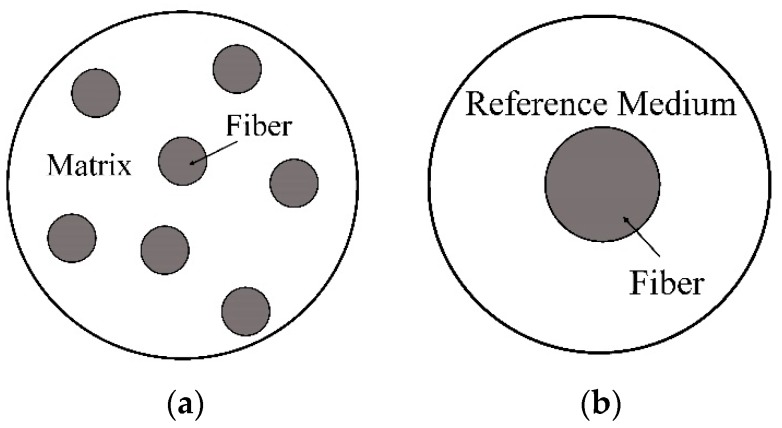
Schematic of a multi-fiber model and a single fiber model. (**a**) Multi-fiber model; (**b**) single fiber model.

The key to a micromechanics model is the determination of a global or local strain/stress concentration tensor. From Equations (5), (6), (12) and (13), it is found that the determination of concentration tensors requires knowledge of the stress and strain fields in the constituent phases of a composite. However, as shown in Figure 6a, due to the interaction between adjacent fibers, it is arduous to obtain an exact stress/strain field of a constituent in a multi-inclusion model. Therefore, a reference medium is introduced, with which the fiber interaction can be approximated in a single fiber model as shown in Figure 6b. The strain and stiffness tensors of the reference medium are denoted by $\varepsilon_{re}$ and $L_{re}$, respectively. A specific definition of the $\varepsilon_{re}$ and $L_{re}$ leads to a specific micromechanics model.

### 3.2. Summary of Elastic Models

#### 3.2.1. Eshelby Model

It was established on a single fiber model (Figure 6b). Let it be subjected to a uniform traction $\sigma_{0}$. Suppose that [167]

(18) $$L_{re}=L_{m}\;\mathrm{and}\;\varepsilon_{re}=\varepsilon_{m}\;$$

where $L_{re}$ and $\varepsilon_{re}$ in Equation (18) stand for the stiffness and strain tensor of the reference medium in Figure 6b, respectively. Since the matrix is infinite, the effect of the fiber on the total strain of the model is neglected, leading to Equation (19).

(19) $$\;\varepsilon =\varepsilon_{m}=M_{m}:\sigma_{0}\;$$

Making use of the Eshelby equivalent inclusion method, the following Equations (20) and (21) are obtained

(20) $$\;\varepsilon_{f}=\varepsilon_{re}+\varepsilon_{pt}=\varepsilon_{m}+S_{m}:\varepsilon^{*}=H_{f}^{ES}:\varepsilon_{m}=H_{f}^{ES}:\varepsilon \;$$

(21) $$\;H_{f}^{ES}={\left[I+S_{m}:M_{m}:\left(L_{f}-L_{m}\right)\right]}^{-1}\;$$

where $\varepsilon_{pt}$ is the perturbed strain tensor due to the presence of the fiber,$\;\varepsilon^{*}$ is an eigenstrain, and $S_{m}$ is an Eshelby tensor. The superscript *ES* designates the Eshelby method. Comparing Equation (5) with (20), the global and the local strain concentration tensors are the same as shown in Equation (22).

(22) $$\;A_{f}=H_{f}^{ES}\;$$

Substituting Equations (21) and (22) into Equation (9), the stiffness tensor of the composite is given by Equation (23).

(23) $$\;L^{ES}=L_{m}+V_{f}\left(L_{f}-L_{m}\right):{\left[I+S_{m}:M_{m}:\left(L_{f}-L_{m}\right)\right]}^{-1}\;$$

Owing to ignoring the interaction of the inclusion with the surrounding fibers, the Eshelby model is applicable only to a composite with a low fiber volume fraction.

#### 3.2.2. SCM

In a SCM [55], the stiffness and strain of the reference medium equal to those of the composite, shown as Equation (24).

(24) $$L_{re}=L\;\mathrm{and}\;\varepsilon_{re}=\varepsilon \;$$

The strain in the fiber is obtained as Equations (25) and (26)

(25) $$\;\varepsilon_{f}=\varepsilon_{re}+\varepsilon_{pt}=\varepsilon +S:\varepsilon^{*}=H_{f}^{SC}:\varepsilon \;$$

(26) $$\;H_{f}^{SC}={\left[I+S:M:\left(L_{f}-L\right)\right]}^{-1}\;$$

where S is the Eshelby tensor from the composite medium. Therefore, Equation (27) is obtained.

(27) $$\;A_{f}=H_{f}^{SC}\;$$

Substituting Equations (25)–(27) into (9), the stiffness tensor of the composite is given by

(28) $$\;L^{SC}=L_{m}+V_{f}\left(L_{f}-L_{m}\right):{\left[I+S:M:\left(L_{f}-L\right)\right]}^{-1}\;$$

Equation (28) is implicit since both sides of it contain the unknown stiffness tensor. Further, the SCM would yield a physical nonsense result for a composite with a rigid or void inhomogeneity [168].

#### 3.2.3. Mori–Tanaka Model

Equation (29) is the assumption of Mori–Tanaka model [56]

(29) $$L_{re}=L_{m}\;\mathrm{and}\;\varepsilon_{re}=\varepsilon_{m}=\varepsilon \;$$

where $\varepsilon =M:\sigma_{0}$. A modification to Equation (20) gives the strain tensor in the fiber as Equations (30) and (31).

(30) $$\;\varepsilon_{f}=\varepsilon_{re}+\varepsilon_{pt}=\varepsilon +S_{m}:\varepsilon^{*}=H_{f}^{MT}:\varepsilon =H_{f}^{MT}:\varepsilon_{m},\;$$

(31) $$\;H_{f}^{MT}={\left[I+S_{m}:M_{m}:\left(L_{f}-L_{m}\right)\right]}^{-1}\;$$

Comparing Equation (12) with (30), one has Equation (32).

(32) $$\;T_{f}=H_{f}^{MT}\;$$

The stiffness tensor in Equation (33) is obtained from Equations (9), (14), (31) and (32) as

(33) $$\begin{array}{c} L^{MT}=L_{m}+V_{f}\left(L_{f}-L_{m}\right):{\left[I+S_{m}:M_{m}:\left(L_{f}-L_{m}\right)\right]}^{-1}\\ :{\left\{V_{m}I+V_{f}{\left[I+S_{m}:M_{m}:\left(L_{f}-L_{m}\right)\right]}^{-1}\right\}}^{-1}\; \end{array}$$

#### 3.2.4. GSCM

**Figure 7:**
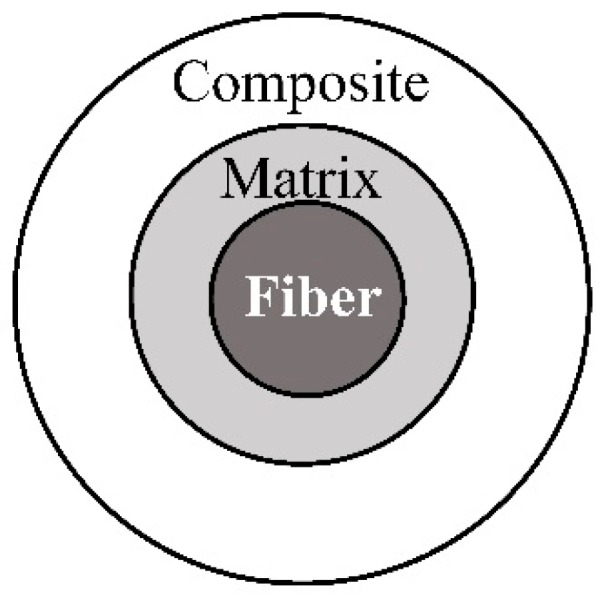
Schematic for generalized self-consistent method (GSCM).

A schematic configuration for the GSCM is shown in Figure 7.

Hill [264] and Hashin [172] presented exact solutions for four of the five effective elastic constants, *E*_11_, $\upsilon_{12}$, *G*_12_, and *E*_22_, as Equations (34)–(38)

(34) $$\;E_{11}=V_{f}E_{11}^{f}+V_{m}E^{m}+\frac{4V_{f}\left(1-V_{f}\right){\left(\upsilon_{12}^{f}-\upsilon^{m}\right)}^{2}}{\frac{V_{f}}{k^{m}}+\frac{1-V_{f}}{k^{m}}+\frac{1}{G^{m}}}\;$$

(35) $$\;\upsilon_{12}=V_{f}\upsilon_{11}^{f}+V_{m}\upsilon^{m}+\frac{V_{f}\left(1-V_{f}\right){\left(\upsilon_{12}^{f}-\upsilon^{m}\right)}^{2}}{\frac{V_{f}}{k^{m}}+\frac{1-V_{f}}{k^{m}}+\frac{1}{G^{m}}}\left(\frac{1}{k^{m}}-\frac{1}{k^{f}}\right)\;$$

(36) $$\;G_{12}=G^{m}\frac{\left(G_{12}^{f}+G^{m}\right)+V_{f}\left(G_{12}^{f}-G^{m}\right)}{\left(G_{12}^{f}+G^{m}\right)-V_{f}\left(G_{12}^{f}-G^{m}\right)}\;$$

(37) $$\;E_{22}=\frac{4}{\frac{1}{k_{22}}+\frac{1}{G_{23}}+\frac{4\upsilon_{12}^{2}}{E_{11}}}\;$$

(38) $$\;k_{22}=k^{m}+\frac{V_{f}}{\frac{1}{k^{f}-k^{m}}+\frac{1-V_{f}}{k^{m}+G^{m}}}\;$$

where k, $k^{f}$, and $k^{m}$ are the transverse bulk moduli of the composite, fiber, and matrix given, respectively, as Equation (39).

(39) $$\;k^{r}=\frac{L_{22}^{r}+L_{23}^{r}}{2},\;r=f,m\;$$

$L_{ij}^{r}$ represents a stiffness component of a constituent material.

As for the transverse shear modulus $G_{23}$, only the upper and lower bounds were provided by Hill [264] and Hashin [172]. Christensen and Lo [171] derived an explicit solution for $G_{23}$ when the composite is made of isotropic fiber and matrix. Luo and Weng [265] obtained the displacement fields in the fiber (*f*), matrix (*m*), and the composite media of Figure 7. Both the fiber and matrix can be transversely isotropic. Luo and Weng’s solutions are shown as Equations (40)–(46).

(40) $$\;u_{r}^{f}=\left[d_{1}r+d_{2}\left(\eta_{f}-3\right)\frac{r^{3}}{a^{2}}\right]cos2\theta \;$$

(41) $$\;u_{\theta }^{f}=\left[-d_{1}r+d_{2}\left(\eta_{f}+3\right)\frac{r^{3}}{a^{2}}\right]sin2\theta \;$$

(42) $$\;u_{r}^{m}=\left[d_{3}r+d_{4}\left(\eta_{m}-3\right)\frac{r^{3}}{b^{2}}+d_{5}\frac{b^{4}}{r^{3}}+d_{6}\left(\eta_{m}+1\right)\frac{b^{2}}{r}\right]cos2\theta \;$$

(43) $$\;u_{\theta }^{m}=\left[-d_{3}r+d_{4}\left(\eta_{m}+3\right)\frac{r^{3}}{b^{2}}+d_{5}\frac{b^{4}}{r^{3}}-d_{6}\left(\eta_{m}-1\right)\frac{b^{2}}{r}\right]sin2\theta \;$$

(44) $$\;u_{r}^{c}=\left[d_{7}\frac{b^{4}}{r^{3}}+d_{8}\left(\eta_{c}+1\right)\frac{b^{2}}{r}\right]cos2\theta \;$$

(45) $$\;u_{\theta }^{c}=\left[d_{7}\frac{b^{4}}{r^{3}}-d_{8}\left(\eta_{c}-1\right)\frac{b^{2}}{r}\right]sin2\theta \;$$

(46) $$\;u_{z}^{i}=0,\;i=f,m,c\;$$

where $d_{i},\;i=1,2\ldots 8$, are unknown coefficients to be solved using the continuity conditions. Once the homogenized stresses and strains of the three-phases are determined from the displacement fields, the shear modulus is obtained.

#### 3.2.5. Rule of Mixture

Owing to its simplicity, the rule of mixture [183,184] is widely used in engineering [266,267,268,269,270]. By this model, the five elastic moduli of the composite are expressed as Equations (47)–(51).

(47) $$\;E_{11}=V_{f}E_{11}^{f}+V_{m}E^{m}\;$$

(48) $$\;\upsilon_{12}=V_{f}\upsilon_{12}^{f}+V_{m}\upsilon^{m}\;$$

(49) $$\;E_{22}=\frac{E^{m}}{1-V_{f}\left(1-\frac{E^{m}}{E_{22}^{f}}\right)}\;$$

(50) $$\;G_{12}=\frac{G^{m}}{1-V_{f}\left(1-\frac{G^{m}}{G_{12}^{f}}\right)}\;$$

(51) $$\;G_{23}=\frac{G^{m}}{1-V_{f}\left(1-\frac{G^{m}}{G_{23}^{f}}\right)}\;$$

#### 3.2.6. Chamis Model

The longitudinal Young’s modulus and the major Poisson ratio in Chamis model [271] coincide with the rule of mixture. The other three moduli are given by Equations (52)–(54).

(52) $$\;E_{22}=\frac{E^{m}}{1-\sqrt{V_{f}}\left(1-\frac{E^{m}}{E_{22}^{f}}\right)}\;$$

(53) $$\;G_{12}=\frac{G^{m}}{1-\sqrt{V_{f}}\left(1-\frac{G^{m}}{G_{12}^{f}}\right)}\;$$

(54) $$\;G_{23}=\frac{G^{m}}{1-\sqrt{V_{f}}\left(1-\frac{G^{m}}{G_{23}^{f}}\right)}\;$$

#### 3.2.7. Halpin–Tsai Equations

As stated by Halpin and Kardos [187], the Halpin–Tsai equations were modified from those of GSCM with some engineering based considerations. The expressions for $E_{11}$ and $\upsilon_{12}$ are consistent with Equations (47) and (48), whereas $E_{22}$ is calculated from Equation (37). Table 2 shows the expressions for the other moduli.

#### 3.2.8. Bridging Model

Huang’s bridging model has the following expressions (Equations (55) and (56)) [188].

(55) $$\;\left\{\sigma_{i}^{m}\right\}=\left[A_{ij}\right]\left\{\sigma_{i}^{f}\right\},\;i,j=1,2\ldots 6\;$$

(56) $$\;\left[M_{ij}\right]=\left(V_{f}\left[M_{ij}^{f}\right]+V_{m}\left[M_{ij}^{m}\right]\left[A_{ij}\right]\right){\left(V_{f}\left[I\right]+V_{m}\left[A_{ij}\right]\right)}^{-1},\;i,j=1,2\ldots 6\;$$

where $\left\{\sigma_{i}^{r}\right\}={\left\{\begin{array}{cc} \begin{array}{ccc} \sigma_{11}^{r} & \sigma_{22}^{r} & \sigma_{33}^{r} \end{array} & \begin{array}{ccc} \sigma_{23}^{r} & \sigma_{13}^{r} & \sigma_{12}^{r} \end{array} \end{array}\right\}}^{T},\;\;r=f,m$ are the homogenized stress vectors of the fiber and matrix. The explicit bridging tensor $\left[A_{ij}\right]$ is as Equations (57)–(60).

(57) $$\;\left[A_{ij}\right]=\left[\begin{array}{cccccc} a_{11} & a_{12} & a_{13} & 0 & 0 & 0\\ 0 & a_{22} & 0 & 0 & 0 & 0\\ 0 & 0 & a_{33} & 0 & 0 & 0\\ 0 & 0 & 0 & a_{44} & 0 & 0\\ 0 & 0 & 0 & 0 & a_{55} & 0\\ 0 & 0 & 0 & 0 & 0 & a_{66} \end{array}\right]$$

(58) $$\;a_{11}=E^{m}/E_{11}^{f}\;$$

(59) $$\;a_{22}=a_{33}=a_{44}=\beta +\left(1-\beta \right)E^{m}/E_{22}^{f},\;0<\beta <1\;$$

(60) $$\;a_{55}=a_{66}=\alpha +\left(1-\alpha \right)G^{m}/G_{12}^{f},\;0<\alpha <1\;$$

β and α are the bridging parameters to better correlate the resulting *E*_22_ and *G*_12_ with experiments. If no experiments are available, β=α=0.3 are mostly recommended. The Equation (61) for $a_{12}$ and $a_{13}$ are solved from the symmetric condition of the composite compliance, i.e., $M_{ij}=M_{ji}$.

(61) $$\;a_{12}=a_{13}=\left(M_{12}^{f}-M_{12}^{m}\right)\left(a_{11}-a_{22}\right)/\left(M_{11}^{f}-M_{11}^{m}\right)\;$$

### 3.3. Quantitative Comparison

A comprehensive quantitative comparison on micromechanics models is desirable both in academic and engineering applications. However, it is almost impossible collect all experiment data in literature for validation. Consider that the WWFE is a worldwide well-known and trustworthy academic activity. Measured elastic properties of the 9 UD composites together with the monolithic fiber and matrix property parameters provided in three world-wide failure exercises (WWFEs) [272,273,274] are used to benchmark the predictions by the different micromechanics models. In addition, three numerical models, i.e., the FEM, FVDAM, and the GMC, are also compared. Please note that all the 9 UD composites are all long fibrous, epoxy matrix, and fiber volume fraction of around 60%. Thus, cautiously speaking, the comparison results are limited to elastic behaviors of long fiber reinforced epoxy matrix with intermediate-high fiber volume fraction.

Since all of the models give essentially the same results for $E_{11}$, only the averaged correlation errors for the other four moduli are shown in Table 3, Table 4, Table 5 and Table 6. Table 7 summarizes the overall averaged errors for the five elastic constants. Information of the 9 composites is given in Appendix A Table A1, whereas detailed predictions by all the models are listed in Table A2, Table A3, Table A4, Table A5, Table A6, Table A7, Table A8, Table A9, Table A10, Table A11 and Table A12.

Please note that the bridging parameters α,β are adjustable according to experiments. Without experiment as reference, α=β=0.3 is recommended. Table 7 shows that bridging model with α=β=0.3 gives the best overall prediction accuracy for elastic behaviors of the 9 UD composites among all the homogenization models involved. In addition, the expressions of the bridging model for homogenized stresses of the fiber and matrix are explicit and the simplest, making it convenient in application. Another advantage of the bridging model is in the bridging parameters, α and β, which are semi-empirical to implicitly represent an effect of some uncertain factors such as random fiber arrangement or imperfect interface on a composite response. Considering the adjustability of α,β, it is worthy to expect that the bridging model may also give satisfactory prediction of elastic properties for other kinds of long fiber composites in addition to the 9 UD ones. Bridging model has been programmed into a general-purpose user-subroutine, Bridging model for analysis of composites (BMANC) [275], which can be combined with an FEM software package such as ABAQUS to analyze linear, nonlinear, failure, and strength behaviors of a complex structure with composites involved.

Chamis model is simple and good in prediction accuracy. The FEM and the FVDAM predictions also agree well with the experimental data. Besides, the numerical models can deal with more complicated fiber-induced geometry such as irregular fiber cross-section or random fiber distribution. Once in a while with no experimental data available, the FEM is used to benchmark other kinds of solutions. The GMC is another kind of numerical model but gives inferior accuracy due to the assumption of uniform strains in cells. Better predictions can be attained by a high fidelity generalized method of cell (HFGMC) [276], but its computational efficiency is lower.

SCM, GSCM, Mori–Tanaka, and Halpin–Tsai give comparable predictions. Amongst, GSCM performed the best. On the other hand, Halpin–Tsai equations and Mori–Tanaka model are more widely used due to their simplicity. It should be noted that the prediction accuracy of $G_{23}$ by SCM is not satisfactory (61% error), although its results for the other four constants are good. For a composite containing rigid or void inhomogeneity, SCM may lead to non-physical $G_{23}$ [168].

The remaining two models, the rule of mixture and Eshelby model, ranked the lowest. The uniform strain/stress assumption used in the rule of mixture violates the continuity condition at fiber/matrix interface, whereas the major error of Eshelby model is resulted from the ignorance of the fiber interaction.

## 4. Comparison on Elastoplastic Behavior

In this work, a comparison of elastoplastic theories is restricted to static, monotonic, and proportional loads. A brief introduction is given on whether a model is applicable to non-monotonic and non-proportional load conditions. Three kinds of UD composites are taken for example in the comparative study, i.e., E-glass/Epoxy, IM7/8551-7, and AS4/Peek UD composites. E-glass is a kind of glass fiber and IM7 and AS4 are carbon fiber. Epoxy and 8551-7 are thermoset while Peek is thermoplastic. Thus, the three composites can reasonably represent most common seen fiber reinforced plastic composites. Regarding the load conditions, we choose transverse compression, in-plane shear, and off-axial tension as examples. It is because the longitudinal tensile/compressive and transverse tensile behaviors of a composite are usually linear elastic. Without consideration of interface damage, the nonlinearity of a composite majorly comes from transverse compression and in-plane shear deformation. In addition, off-axial tensile can be seen as a combination of transverse tension and in-plane shear, representing a kind of multi-axial load case. Thus, we choose the three kinds of load cases as benchmark.

**Figure 8:**
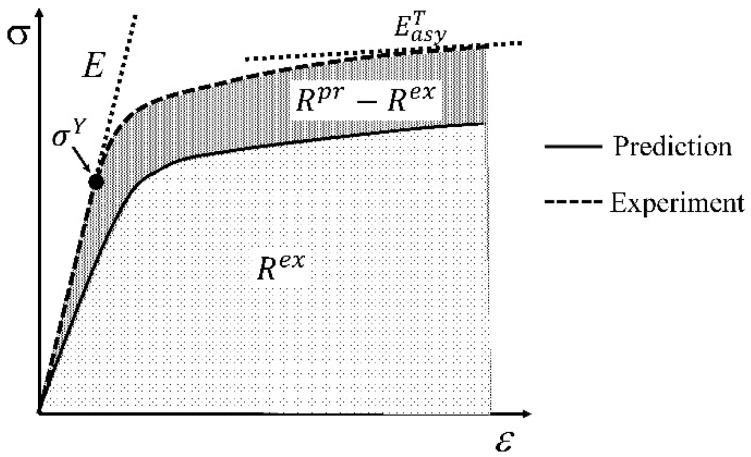
Illustration of a typical elastoplastic stress-strain curve.

A rule is needed to judge the efficiency of different models for the predicted elastoplastic responses. Let us choose three parameters. They are the elastic modulus E or G, the yield point $\sigma^{Y}$, and the asymptotic tangent modulus $E_{asy}^{T}$ or $G_{asy}^{T}$ as schematically shown in Figure 8. The latter one, $E_{asy}^{T}$ or $G_{asy}^{T}$, is defined as the minimum value of the tangents to the tensile or shear stress strain curve of a material [57,95]. A predicted elastic constant affects the prediction accuracy of the stress-strain curve in the elastic part. The evaluation of the yield point determines when a material yields. In fact, the slope variation of the stress strain curve depends on the predicted yield point. In other words, the reduction rate of a predicted modulus is controlled by the yield point. The asymptotic tangent modulus $E_{asy}^{T}$ or $G_{asy}^{T}$ is a new concept introduced in this work. It is defined as the minimum value of the tangents to the tensile or shear stress strain curve of a material. As shown in Equation (62), a tangential modulus is negatively correlated to a plastic strain. Thus, the asymptotic tangent modulus can partly reflect how much plastic strain a material can bear.

(62) $$\;d\varepsilon^{p}=-d\sigma /E+d\sigma /E^{T}\;$$

In addition to the E or G, $\sigma^{Y}$, and $E_{asy}^{T}$ or $G_{asy}^{T}$, an overall error, $ER^{ov}$ as shown in Equation (63), is introduced to characterize the overall prediction performance of a model.

(63) $$\;ER^{ov}=\frac{1}{n}\overset{n}{\underset{i}{\sum }}Abs\left(\frac{R^{pr}-R^{ex}}{R^{ex}}\right)\times 100\%$$

where *n* is the amount of available experiment curves. As shown in Figure 8, $R^{pr}$ is the enclosed area between the predicted curve and strain-axis, and $R^{ex}$ is the experimental one. For a uniaxial tension/compression or pure shear stress state, the enclosed area corresponds to the accumulated strain energy. For a complex stress state, the area corresponds to a part of the strain energy. Note that $ER^{ov}$ can only reflect one valuable side view of a model’s capability. When choosing a model, readers should comprehensively evaluate the applicability of a model from their own viewpoint.

### 4.1. Comparison on Micromechanics Models

Among the eight analytical models, Mori–Tanaka method, SCM, bridging model, and Chamis model have been widely used in the analysis of an elastoplastic response with the help of a linearization. For consistency purpose, only the first-moment von-Mises equivalent stress (Section 4.2.1) and the tangent linearization (Section 4.3.2) are incorporated. It should be noted that, for Mori–Tanaka model, bridging model, and self-consistent model, the elastoplastic stiffness of a matrix and the corresponding Eshelby tensor can be anisotropic. However, for Chamis model, the elastoplastic stiffness of a matrix is pre-assumed to be isotropic. Thus, from the viewpoint of consistency, the stiffness of a matrix is isotropicalized (Section 4.4.2) for all the four models. An instantaneous stiffness tensor of the composite by SCM and Mori–Tanaka model are given as Equations (64) and (65) [194,277].

(64) $$\;L_{SC}^{tan}=L_{m}^{tan}+V_{f}\left(L_{f}-L_{m}^{tan}\right):{\left[I+S_{\;}^{tan}:M_{SC}^{tan}:\left(L_{f}-L_{SC}^{tan}\right)\right]}^{-1}\;$$

(65) $$\begin{array}{c} L_{MT}^{tan}=L_{m}^{tan}+V_{f}\left(L_{f}-L_{m}^{tan}\right):{\left[I+S_{m}^{tan}:M_{m}^{tan}:\left(L_{f}-L_{m}^{tan}\right)\right]}^{-1}\\ :{\left\{V_{m}I+V_{f}{\left[I+S_{m}^{tan}:M_{m}^{tan}:\left(L_{f}-L_{m}^{tan}\right)\right]}^{-1}\right\}}^{-1}\; \end{array}$$

where *tan* indicates the instantaneous quantity in a tangent form. The subscripts *SC* and *MT* denote the quantities from SCM and Mori–Tanaka model, respectively.

Regarding bridging model, owing to coupling between normal and shear stresses in a plastic deformation, and elastoplastic bridging tensor is modified to Equations (66)–(69) [278].

(66) $$\left[A_{ij}^{ep}\right]=[\begin{array}{cccccc} a_{11}^{ep} & a_{12}^{ep} & a_{13}^{ep} & a_{14}^{ep} & a_{15}^{ep} & a_{16}^{ep}\\ 0 & a_{22}^{ep} & a_{23}^{ep} & a_{24}^{ep} & a_{25}^{ep} & a_{26}^{ep}\\ 0 & 0 & a_{33}^{ep} & a_{34}^{ep} & a_{35}^{ep} & a_{36}^{ep}\\ 0 & 0 & 0 & a_{44}^{ep} & a_{45}^{ep} & a_{46}^{ep}\\ 0 & 0 & 0 & 0 & a_{55}^{ep} & a_{56}^{ep}\\ 0 & 0 & 0 & 0 & 0 & a_{66}^{ep} \end{array}]$$

(67) $$\;a_{11}^{ep}=E_{m}^{tan}/E_{11}^{f}\;$$

(68) $$\;a_{22}^{ep}=a_{33}^{ep}=a_{33}^{44}=\beta +\left(1-\beta \right)E_{m}^{tan}/E_{22}^{f}\;$$

(69) $$\;a_{55}^{ep}=a_{66}^{ep}=\alpha +\left(1-\alpha \right)G_{m}^{tan}/G_{12}^{f}\;$$

The superscript “*ep*” represents elastoplastic. The off diagonal elements are solved from the condition that the instantaneous compliance tensor of the composite, i.e., $M_{ij}^{tan}=M_{ji}^{tan}$, is symmetric. $M_{ij}^{tan}$ is given by Equation (70).

(70) $$\;\left[M_{ij}^{tan}\right]=\left(V_{f}\left[M_{ij}^{f}\right]+V_{m}\left[M_{ij}^{m-tan}\right]\left[A_{ij}^{ep}\right]\right){\left(V_{f}\left[I\right]+V_{m}\left[A_{ij}^{ep}\right]\right)}^{-1},\;i,j=1,2\ldots 6\;$$

As for an elastoplastic Chamis model, Equation (71)–(75) are applied [271]:

(71) $$\;E_{11}^{tan}=V_{f}E_{11}^{f}+V_{m}E_{m}^{tan}\;$$

(72) $$\;\upsilon_{12}^{tan}=V_{f}\upsilon_{12}^{f}+V_{m}\upsilon_{m}^{tan}\;$$

(73) $$\;E_{22}^{tan}=\frac{E_{m}^{tan}}{1-\sqrt{V_{f}}\left(1-\frac{E_{m}^{tan}}{E_{22}^{f}}\right)}\;$$

(74) $$\;G_{12}^{tan}=\frac{G_{m}^{tan}}{1-\sqrt{V_{f}}\left(1-\frac{G_{m}^{tan}}{G_{12}^{f}}\right)}\;$$

(75) $$\;G_{23}^{tan}=\frac{G_{m}^{tan}}{1-\sqrt{V_{f}}\left(1-\frac{G_{m}^{tan}}{G_{23}^{f}}\right)}\;$$

**Figure 9:**
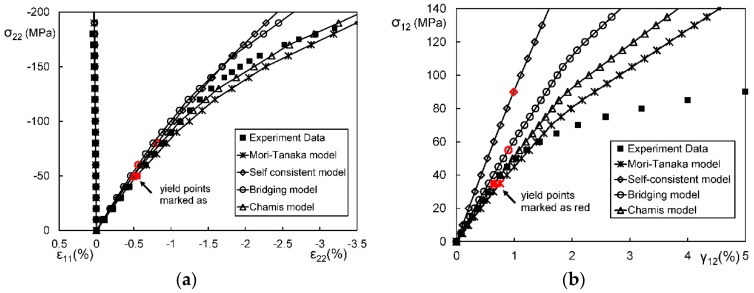
Comparison between elastoplastic models (IM7/8551-7 UD composite). (**a**) Transverse compression; (**b**) In-plane shear.

**Figure 10:**
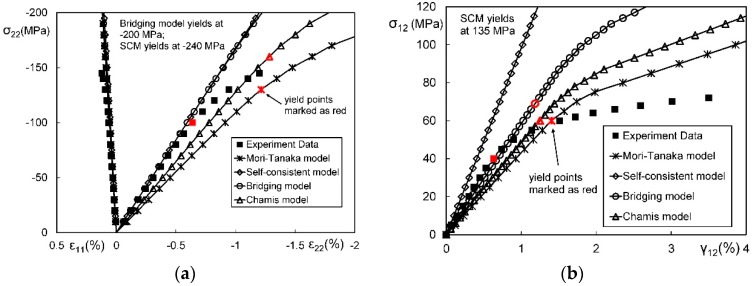
Comparison between elastoplastic models (E-Glass/Epoxy UD composite). (**a**) Transverse compression; (**b**) In-plane shear.

**Figure 11:**
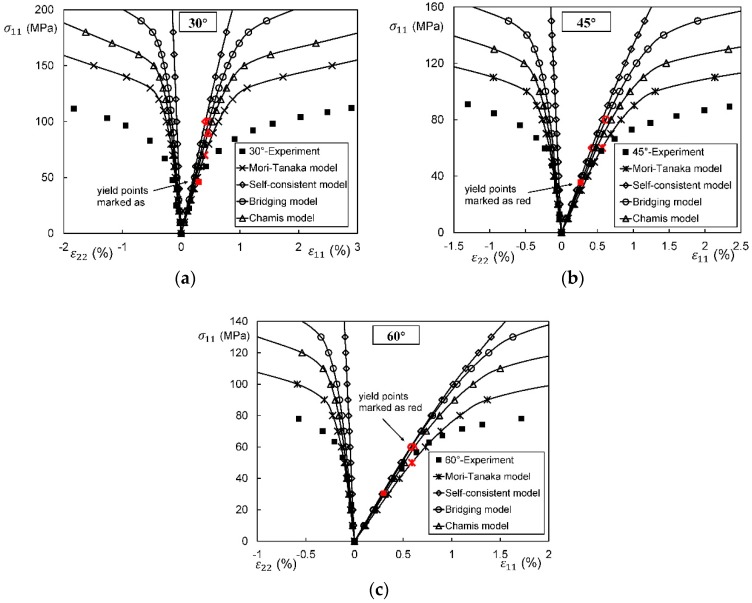
Comparison between elastoplastic models (AS4/Peek UD composite). (**a**) 30° off-axis tension; (**b**) 45° off-axis tension; (**c**) 60° off-axis tension.

In general, an instantaneous compliance tensor of the matrix in an elastoplastic Mori–Tanaka model, SCM, and bridging model is anisotropic defined by a classical flow rule. However, an elastoplastic Chamis model is only applicable to cases where the matrix can undergo an isotropic deformation up to failure. Figure 9, Figure 10 and Figure 11 are the elasto-plastic stress-strain curves of three kinds of UD composites predicted by the four elastoplastic models mentioned above. The experimental data are taken from Kaddour and Hinton [273] and Kawai et al. [279].

From Figure 9, Figure 10 and Figure 11 and Table 8, it is found that the prediction results of all the four models are too stiff. The yield points and asymptotic tangent moduli provided by the SCM are significantly overestimated for all the cases in Figure 9, Figure 10 and Figure 11. It is because that, in the configuration of the SCM, a fiber is directly surrounded by a composite medium. Since the composite is much stiffer than the constituent matrix, the plastic deformation of the matrix is underestimated, leading to overestimated stiffness of the composite. In elastic range, the bridging model is recommended. However, regarding yielding, asymptotic modulus and the overall error, all the four model are not satisfactory. More attempts should be made on modifying the prediction of the yield points and the asymptotic tangent moduli. In addition to a prediction accuracy, the capability on dealing with a tension-shear stress coupling is also critical. From this point, the Chamis model is inferior to the other three models.

It is necessary to point out that the comparisons in Figure 9, Figure 10 and Figure 11 are based on the assumption of an isotropic elastoplastic matrix and the tangent linearization, whereas other factors such as the stress fluctuations and the SCFs are not accounted for. In addition, the averaged error data in Table 8 is useful for readers to evaluate a model but insufficient to tell which model is better. Cautiously speaking, the comparison results are valid only for long fiber reinforced plastic composites with intermediate fiber volume fraction under static, monotonic, and proportional load conditions.

### 4.2. Comparison on Modifications on Yield Stress

As mentioned in Section 4.1, the accuracy in evaluation of a yield point is critical to the prediction capability of an elastoplastic model. A von-Mises equivalent stress is employed to detect yielding. Generally, the equivalent stress is calculated based on the first moment stress (homogenized stress) of a material. However, it is reported that the first moment approach cannot reflect a stress fluctuation, leading to an underestimation of an equivalent stress [205,222,223]. Thus, a second-moment approach is developed [205,222,223]. On the other hand, Huang [103,108,109] pointed out that the homogenized stresses of the matrix in a composite must be converted into true values before an effective property of the composite is evaluated in terms of the fiber and matrix’s original property parameters. The conversion is done by multiplying the homogenized quantities with the respective SCFs of the matrix in the composite. Using the true stress concept, the prediction on a yield behavior of a composite can also be significantly improved.

In this section, the tangent linearization is applied to the three approaches, i.e., first moment, second moment, and SCFs. Both the elastoplastic matrix and Eshelby tensor are anisotropic, which are given in Section 4.3.2.

#### 4.2.1. First Moment Approach

For the first moment approach, the J2 flow rule is given as Equations (76)–(78) [114].

(76) $$\;f\left(\sigma_{eq},\sigma_{Y}\right)=\frac{1}{3}{\left(\sigma_{eq}^{1st}\right)}^{2}-\frac{1}{3}\sigma_{Y}^{2},\{\begin{array}{l} f\left(\sigma_{eq},\sigma_{Y}\right)=0,\;yield\\ f\left(\sigma_{eq},\sigma_{Y}\right)<0,\;not\;yield \end{array}\;$$

(77) $$\;\sigma_{eq}^{1st}=\sqrt{\frac{3}{2}\sigma^{\prime }:\sigma^{\prime }}=\sqrt{\frac{3}{2}I^{dev}∷\sigma ⊗\sigma }\;$$

(78) $$\;I^{dev}=\frac{1}{2}\delta_{ik}\delta_{jl}+\frac{1}{2}\delta_{il}\delta_{jk}-\frac{1}{3}\delta_{ij}\delta_{kl}\;$$

where $\sigma_{eq}^{1st}$ is the von-Mises equivalent stress based on the first moment approach. $\sigma_{Y}$ is the yield strength, depending on a work-hardening behavior of a material. The symbol ⊗ denotes a tensor product. $I^{dev}$ is a fourth-order deviatoric tensor. σ and $\sigma^{\prime }$ are, respectively, the homogenized stresses and the corresponding deviatoric of a material. The key point of the first moment approach is that the von-Mises equivalent stress is calculated from a homogenized stress σ or $\sigma^{\prime }$ other than the point-wise one σ(x) or $\sigma^{\prime }\left(x\right)$.

#### 4.2.2. Second-Moment Approach

The core concept of the second-moment approach is that a von-Mises equivalent stress is firstly evaluated point-wisely. Then a homogenized von-Mises stress is calculated by volume averaging the obtained point-wise von-Mises stresses (see Equation (79)).

(79) $$\;\sigma_{eq}^{2nd}=\sqrt{\frac{3}{2}I^{dev}∷{\langle \sigma \left(x\right)⊗\sigma \left(x\right)\rangle }_{r}}\;$$

In Equation (79), $\sigma_{eq}^{2nd}$ means a von-Mises equivalent stress based on a second-moment approach. The bracket ${\langle \cdot \rangle }_{r}$ represents a volume average manipulation of a phase *r*⊗ given by Equation (80).

(80) $${\langle \sigma \left(x\right)⊗\sigma \left(x\right)\rangle }_{r}=\frac{1}{V_{r}}\sigma_{r}:\frac{\partial M}{\partial M_{r}}:\sigma_{r}\;$$

where $M_{r},\;r=f,m$*,* are the elastic compliance tensors of fiber and matrix, respectively. M is the effective elastic compliance tensor of the composite calculated from a selected homogenization.

**Figure 12:**
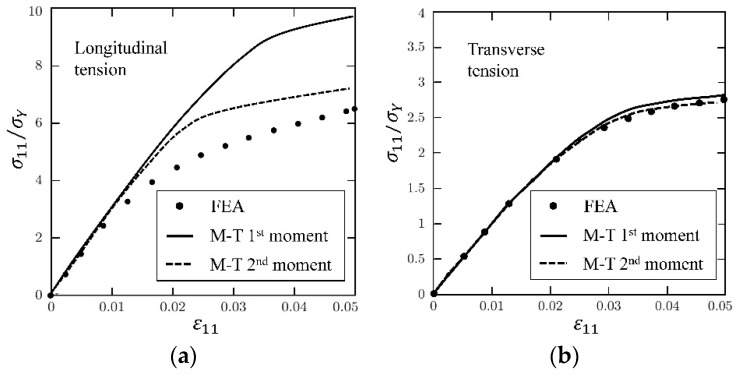
Short fiber reinforced polyamide composite under uniaxial tension [280]. (**a**) Longitudinal tension; (**b**) Transverse tension.

**Figure 13:**
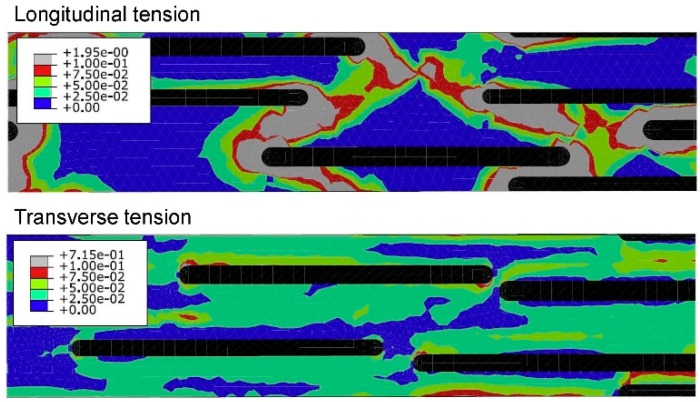
Finite element analysis (FEA) strain contour for short fiber reinforced polyamide composite [280].

**Figure 14:**
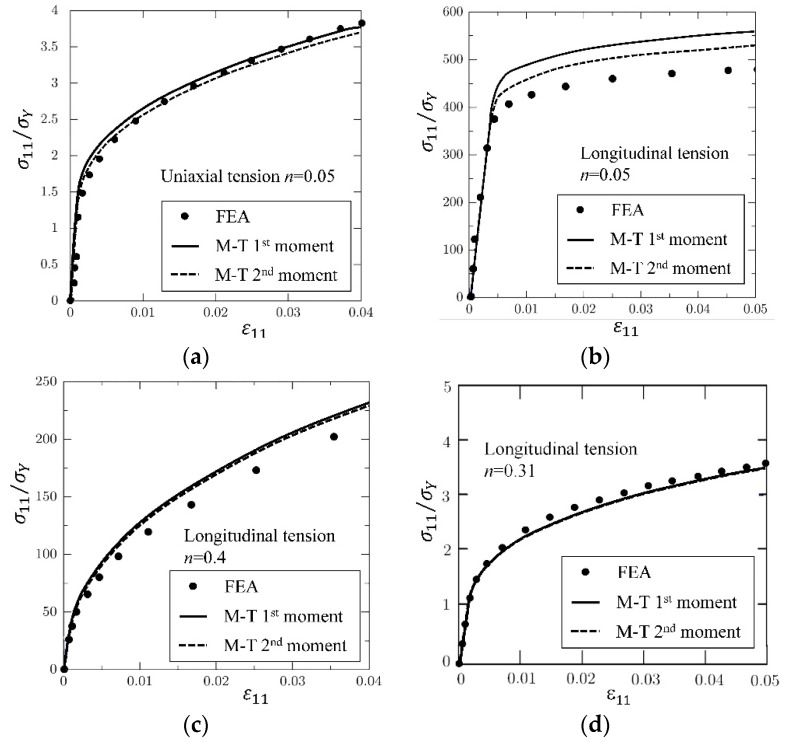
Comparison between the first and second-moment approach. (**a**) Ceramic reinforced aluminum composite (aspect ratio = 1, plastic parameter *n* = 0.05); (**b**) Ceramic reinforced aluminum composite (aspect ratio = 3, plastic parameter *n* = 0.05); (**c**) Ceramic reinforced aluminum composite (aspect ratio = 3, plastic parameter *n* = 0.4); (**d**) two-phase steel with martensite inclusions (aspect ratio = 3, plastic parameter *n* = 0.31).

As addressed by Suquet [223] and Hu [222], the second-moment approach contributes more when the stress/strain fields variation is significant. Theoretically, the field variation in a short fibrous composite is more obvious than that in a long fibrous one. Thus, short fibrous composite is taken as illustration in the comparison between the first and second moment approaches. Doghri et al. [280] conducted a comprehensive study on the second-moment approach based on a kind of aligned short fibrous composite. Figure 12, Figure 13 and Figure 14 are taken from their work. M-T 1st and 2nd represent Mori–Tanaka model based on the first and second-moment approaches, respectively.

Figure 12 are stress-strain curves of an aligned short fibrous composite under a longitudinal/ transverse tensile load. From Figure 12, it is found that the second-moment approach can significantly improve the prediction accuracy for a short fiber reinforced polyamide composite under longitudinal tension but makes a low contribution for the case of transverse tension. In order to investigate the reason, FEM plastic strain gradient contours for the composite under the two load cases are plot in Figure 13. It is shown that the strain gradient in the longitudinal tension is much more noticeable than that in the transverse one. The results validate the conclusion of Suquet [223] and Hu [222] that the second-moment approach can make more contributions for cases with significant stress or strain gradients. Please note that the fiber configurations in Figure 13 are not exactly coincident. Theoretically, strain concentration becomes more obviously when the ends of two fibers get closer. But in the second image of Figure 13, the strain distribution is much more homogeneous even at the center-right point where two fiber ends are close to each other. Thus, compared with two coincident images, the images in Figure 13 are more convictive on illustrating the effect of loads on strain distributions. A further comparison is made in Figure 14 to investigate in which situation a second-moment approach is necessary [280]. Doghri et al. summarized three conditions [280]:

(a) the inclusion aspect ratio is larger than 1,
(b) the elastic stiffness contrast between a fiber and a matrix is high,
(c) work-hardening phenomenon of a matrix is not significant.

When the conditions are satisfied, an application of the second-moment approach can make a significant improvement.

#### 4.2.3. SCFs in Matrix

A literature has shown [271] that a predicted matrix-failure controlled strength of a fibrous composite especially under a transverse tension based on the homogenized stresses of the matrix is much lower than the measured counterpart. Liu and Huang [101] pointed out that this phenomenon is resulted from an ignorance of the stress concentrations in the matrix due to introduction of the fiber. They believed that this problem could be tackled by introducing the SCFs to modify the homogenized matrix stresses. The concept of the SCFs in the matrix is originally proposed for a failure prediction based on a bridging model. But it can also be used in an elastoplastic model to estimate the yield behavior of the composite. The SCFs in the matrix of a composite under transverse tension, $K_{22}^{t}$, and compression, $K_{22}^{c}$, are given as Equations (81)–(86) [109].

(81) $$\begin{array}{c} \;K_{22}\left(\varphi \right)=\{1+\frac{a_{1}}{2}\sqrt{V_{f}}cos2\varphi +\frac{b_{1}}{2\left(1-\sqrt{V_{f}}\right)}[V_{f}^{2}cos4\varphi +4V_{f}{\left(cos\varphi \right)}^{2}\left(1-2cos2\varphi \right)\\ \;+\sqrt{V_{f}}\left(2cos2\varphi +cos4\varphi \right)]\}\frac{\left(V_{f}+0.3V_{m}\right)E_{22}^{f}+0.7V_{m}E^{m}}{0.3E_{22}^{f}+0.7E^{m}}\; \end{array}$$

(82) $$\;a_{1}=\frac{2E_{22}^{f}E^{m}{\left(\upsilon_{12}^{f}\right)}^{2}+E_{11}^{f}\left\{E^{m}\left(\upsilon_{23}^{f}-1\right)-E_{22}^{f}\left[2{\left(\upsilon^{m}\right)}^{2}+\upsilon^{m}-1\right]\right\}}{E_{11}^{f}\left[E_{22}^{f}+E^{m}\left(1-\upsilon_{23}^{f}\right)+E_{22}^{f}\upsilon^{m}\right]-2E_{22}^{f}E^{m}{\left(\upsilon_{12}^{f}\right)}^{2}}\;$$

(83) $$\;b_{1}=\frac{E^{m}\left(1+\upsilon_{23}^{f}\right)-E_{22}^{f}\left(1+\upsilon^{m}\right)}{E_{22}^{f}\left[4{\left(\upsilon^{m}\right)}^{2}+\upsilon^{m}-3\right]-E^{m}\left(1+\upsilon_{23}^{f}\right)}\;$$

(84) $$\;K_{22}^{t}=K_{22}\left(0\right)\;$$

(85) $$\;K_{22}^{c}=K_{22}\left(\phi \right)\;$$

(86) $$\;\phi =\frac{\pi }{4}+\frac{1}{2}arcsin\frac{\sigma_{u,c}^{m}-\sigma_{u,t}^{m}}{2\sigma_{u,c}^{m}}\;$$

where $\sigma_{u,t}^{m}$ and $\sigma_{u,c}^{m}$ are the tensile and compressive strengths of the matrix, respectively. Owing to transversely isotropic, the SCFs of the matrix in the UD composite along axis 3 are the same as those in the 2nd axis, i.e., $K_{22}=K_{33}$, if the transverse stresses of the matrix, $\sigma_{22}^{m}$ and $\sigma_{33}^{m}$, do not occur simultaneously.

The transverse shear SCF, $K_{23}$, of the matrix is defined according to the Mohr’s rule as Equation (87).

(87) $$\;K_{23}=2\sigma_{u,s}^{m}\sqrt{\frac{K_{22}^{t}K_{22}^{c}}{\sigma_{u,t}^{m}\sigma_{u,c}^{m}}}\;$$

where $\sigma_{u,s}^{m}$ is the shear strength of a matrix.

A matrix SCF under in-plane shear is given by Equations (88) and (89),

(88) $$\;K_{12}=K_{13}=\left[1-V_{f}\frac{G_{12}^{f}-G^{m}}{G_{12}^{f}+G^{m}}\left(W\left(V_{f}\right)-\frac{1}{3}\right)\right]\frac{\left(V_{f}+0.3V_{m}\right)G_{12}^{f}+0.7V_{m}G^{m}}{0.3E_{22}^{f}+0.7E^{m}}\;$$

(89) $$\;W\left(V_{f}\right)\approx \pi \sqrt{V_{f}}\left(-\frac{5}{4096}V_{f}^{2}-\frac{1}{256}V_{f}+\frac{1}{4V_{f}}-\frac{1}{32}\right)\;$$

Then, the matrix stress can be modified as Equations (90) and (91).

(90) $$\;\sigma_{m}^{SCF}=\left\{\begin{array}{cc} \begin{array}{ccc} \sigma_{11}^{m} & K_{22}\sigma_{22}^{m} & K_{33}\sigma_{33}^{m} \end{array} & K_{23}\begin{array}{ccc} \sigma_{23}^{m} & K_{12}\sigma_{13}^{m} & K_{12}\sigma_{12}^{m} \end{array} \end{array}\right\}\;$$

(91) $$K_{22}=\{\begin{array}{c} K_{22}^{t},\sigma_{22}^{m}\geq 0\;\\ K_{22}^{c},\sigma_{22}^{m}<0 \end{array},\;K_{33}=\{\begin{array}{c} K_{33}^{t},\sigma_{33}^{m}\geq 0\;\\ K_{33}^{c},\sigma_{33}^{m}<0 \end{array}\;$$

**Figure 15:**
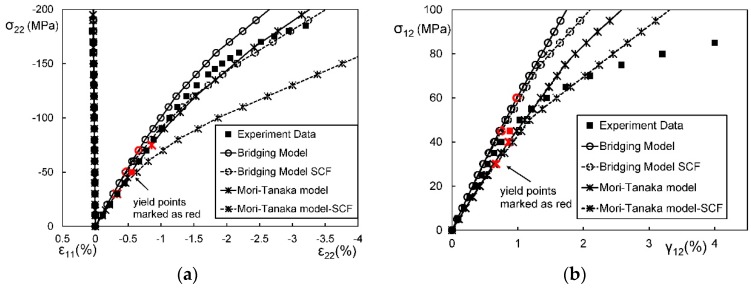
Comparison of models with/without stress concentration factors (SCFs)—IM7/8551-7 UD composite. (**a**) Transverse compression; (**b**) In-plane shear.

**Figure 16:**
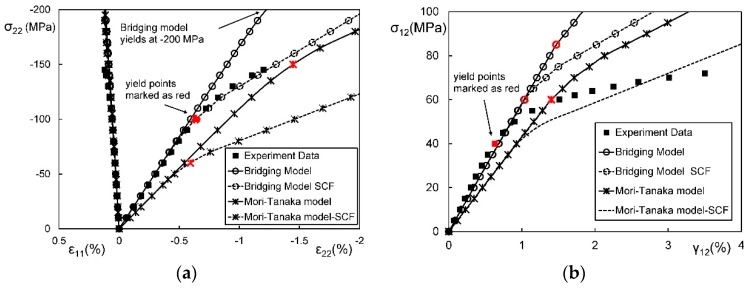
Comparison of models with/without stress concentration factors (SCFs)—E-glass/Epoxy UD composite. (**a**) Transverse compression; (**b**) In-plane shear.

**Figure 17:**
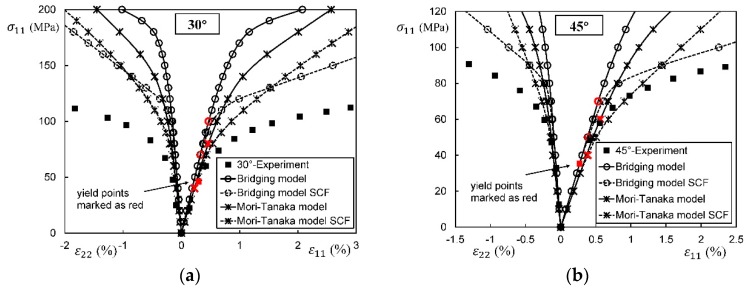

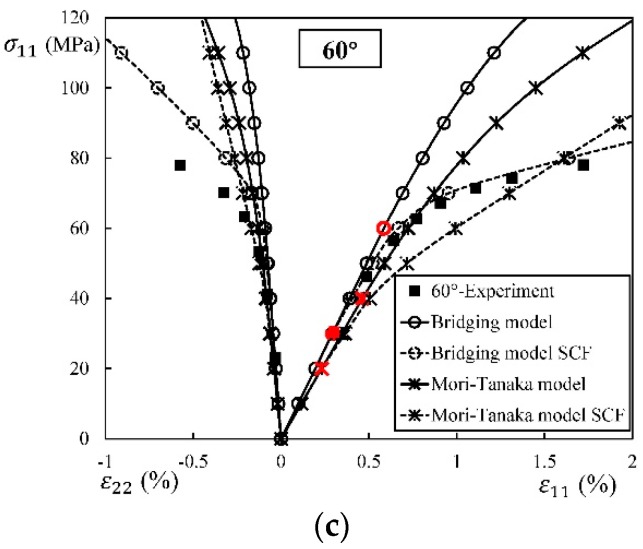
Comparison of models with/without SCFs—AS4/Peek UD composite. (**a**) 30° Off-axis tension; (**b**) 45° Off-axis tension; (**c**) 60° Off-axis tension.

A comparison is made in Figure 15, Figure 16 and Figure 17 between the bridging and Mori–Tanaka models with and without the SCFs. The tangent linearization is employed. The involved elastoplastic compliance/stiffness and the corresponding Eshelby’s tensors are anisotropic. As mentioned in Section 4, the prediction accuracy of an elastoplastic model depends on three aspects, the elastic modulus, the yield stress, and the asymptotic tangent modulus. From Figure 15, Figure 16 and Figure 17, it is seen that the prediction error of the bridging model mainly resulted from the overestimated yield stress and the asymptotic tangent modulus. For the cases shown in Figure 15a, Figure 16a and Figure 17, the prediction accuracies are improved significantly by incorporation of the SCFs. However, for the in-plane shear cases, Figure 15b and Figure 16b, the prediction accuracy is still not satisfactory. It is because the introduction of SCFs can modify the prediction on yield stress but cannot reduce errors coming from the overestimated $G_{asy}^{T}$.

The prediction errors of the Mori–Tanaka model come from all the three aspects, the *E* or *G*, the $\sigma^{Y}$, and the $E_{asy}^{T}$ or $G_{asy}^{T}$. For most cases, the introduction of SCFs can improve its prediction results. But for some cases, the prediction accuracy becomes worse. It is because the yield stress is underestimated by the Mori–Tanaka model with SCFs. In addition, the errors from the overestimated $E_{asy}^{T}$ or $G_{asy}^{T}$ cannot be modified by introducing the SCFs, similarly as the bridging model.

### 4.3. Comparison on Linearization

**Figure 18:**
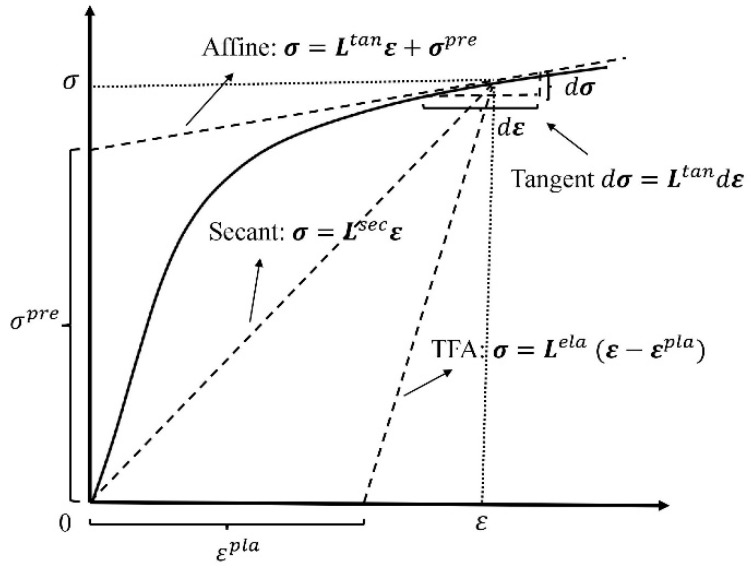
Schematic of linearization theories.

The next step to build an elastoplastic model is in selection of a linearization. For simplicity, only the matrix is treated as elastoplastic while the fiber keeps elastic. Figure 18 shows a schematic of the four most widely used linearization schemes, i.e., the secant linearization, the tangent linearization, the TFA, and the affine formulations. The latest linearization, an incremental-secant scheme, is also introduced later in this section. The Mori–Tanaka model and the first-moment equivalent stress are employed for all the five linearizations.

#### 4.3.1. Secant Linearization

On the secant linearization, it is assumed that the total stresses and strains can be correlated by a “secant” stiffness or compliance tensor through Equations (92) and (93).

(92) $$\varepsilon =M^{sec}\sigma ,\;\sigma =L^{sec}\varepsilon ,$$

(93) $$\varepsilon_{r}=M_{r}^{sec}\sigma_{r},\;\sigma_{r}=L_{r}^{sec}\varepsilon_{r},\;r=f,m\;$$

where the superscript *sec* represents instantaneous quantities in a secant form. The global strain concentration tensor can also be rewritten in a secant form as Equation (94).

(94) $$\;\sigma_{f}=B_{f}^{sec}\sigma ,\;$$

The effective secant stiffness tensor is given by Equation (95).

(95) $$\;M^{sec}=M_{m}^{sec}+V_{f}\left(M_{f}^{sec}-M_{m}^{sec}\right)B_{f}^{sec}\;$$

If the Mori–Tanaka model is employed, the $B_{f}^{sec}$ is obtained as Equations (96) and (97).

(96) $$\;B_{f}^{sec}=P_{f}^{sec}{\left[V_{m}I+V_{f}P_{f}^{sec}\right]}^{-1},\;$$

(97) $$\;P_{f}^{sec}=M_{f}^{-1}{\left[I+S_{m}^{sec}M_{m}^{sec}\left(M_{f}^{-1}-M_{m}^{sec}{}^{-1}\right)\right]}^{-1}M_{m}^{sec}\;$$

The non-zero elements of the $M_{m}^{sec}$ are given by Equations (98)–(100).

(98) $$\;M_{1111}^{m-sec}=M_{2222}^{m-sec}=M_{3333}^{m-sec}=\frac{1}{E_{m}^{sec}}\;$$

(99) $$\;M_{1122}^{m-sec}=M_{2233}^{m-sec}=M_{3311}^{m-sec}=M_{1133}^{m-sec}=M_{2211}^{m-sec}=M_{3322}^{m-sec}=\frac{-\upsilon_{m}^{sec}}{E_{m}^{sec}}\;$$

(100) $$\;M_{1212}^{m-sec}=M_{2323}^{m-sec}=M_{3131}^{m-sec}=\frac{1}{G_{m}^{sec}}\;$$

where $E_{m}^{sec}$, $G_{m}^{sec}$, and $\upsilon_{m}^{sec}$ are the “secant” elastic constants. The secant Young’s modulus $E_{m}^{sec}$ is directly obtained from a given uniaxial tension or compression stress-strain curve. Supposing that the bulk modulus keeps elastic, the secant Poisson’s ratio and the secant shear modulus are obtained, respectively, as Equations (101) and (102).

(101) $$\;\upsilon_{m}^{sec}=\frac{1}{2}-\left(\frac{1}{2}-\upsilon_{m}\right)\frac{E_{m}^{sec}}{E_{m}}\;$$

(102) $$\;G_{m}^{sec}=\frac{E_{m}^{sec}}{2\left(1+\upsilon_{m}^{sec}\right)}\;$$

Equations (103)–(105) list non-zero elements of the “secant ” Eshelby tensor $S_{m}^{sec}$.

(103) $$\;S_{1111}^{m-sec}=S_{2222}^{m-sec}=S_{3333}^{m-sec}=\frac{7-5\upsilon_{m}^{sec}}{15\left(1-\upsilon_{m}^{sec}\right)}\;$$

(104) $$\;S_{1122}^{m-sec}=S_{2233}^{m-sec}=S_{3311}^{m-sec}=S_{1133}^{m-sec}=S_{2211}^{m-sec}=S_{3322}^{m-sec}=\frac{5\upsilon_{m}^{sec}-1}{15\left(1-\upsilon_{m}^{sec}\right)}\;$$

(105) $$\;S_{1212}^{m-sec}=S_{2323}^{m-sec}=S_{3131}^{m-sec}=\frac{4-5\upsilon_{m}^{sec}}{15\left(1-\upsilon_{m}^{sec}\right)}\;$$

It should be noted that Equations (103)–(105) only give the expressions of $S_{m}^{sec}$ for cylindrical fiber reinforced composites. For another shape of the inhomogeneity, the $S_{m}^{sec}$ can also be obtained directly by replacing the elastic properties of the matrix in an elastic Eshelby tensor with the corresponding secant ones.

#### 4.3.2. Tangent Linearization

Equations (106)–(108) are derived from a tangent linearization:

(106) $$d\varepsilon =M^{tan}d\sigma ,\;d\sigma =L^{tan}d\varepsilon \;$$

(107) $$d\varepsilon_{r}=M_{r}^{tan}d\sigma_{r},\;d\sigma_{r}=L_{r}^{tan}d\varepsilon_{r},\;r=f,m\;$$

(108) $$\;d\sigma_{r}=B_{r}^{tan}d\sigma ,\;r=f,m\;$$

where dε, $d\varepsilon_{r}$ and dσ, $d\sigma_{r}$ are the strain and stress tensors of the composite, and the constituent phases, respectively. The effective compliance tensor is derived as Equation (109).

(109) $$\;M^{tan}=M_{m}^{tan}+V_{f}\left(M_{f}^{tan}-M_{m}^{tan}\right)B_{f}^{tan}\;$$

Also, if Mori–Tanaka model is employed, the $B_{f}^{tan}$ is obtained as Equations (110) and (111).

(110) $$\;B_{f}^{tan}=P_{f}^{tan}{\left[V_{m}I+V_{f}P_{f}^{tan}\right]}^{-1}\;$$

(111) $$\;P_{f}^{tan}=M_{f}^{-1}{\left[I+S_{m}^{tan}M_{m}^{tan}\left(M_{f}^{-1}-M_{m}^{tan}{}^{-1}\right)\right]}^{-1}M_{m}^{tan}\;$$

The superscript *tan* represents tangent quantities. Several approaches can be used to determine the $M_{m}^{tan}$ and $S_{m}^{tan}$. In this section, only the most general one is highlighted. When a material undergoes an elasto-plastic deformation, its mechanical properties become anisotropic. If a J2 flow rule is employed, the tangent instantaneous compliance tensor of a matrix is given as Equations (112)–(114) [9]:

(112) $$\;M_{m}^{tan}=M_{m}^{\;}+M_{m}^{pla}\;$$

(113) $$\;M_{m}^{pla}=\frac{9}{4E_{m}^{p}\sigma_{eq}^{2}}\sigma^{\prime }⊗\sigma^{\prime }\;$$

(114) $$\;E_{m}^{p}=\frac{E_{m}E_{m}^{tan}}{E_{m}-E_{m}^{tan}}\;$$

$M_{m}^{\;}$ is the elastic compliance tensor of a matrix. $\sigma_{eq}$ is an von-Mises equivalent stress given in Equation (77). $\sigma^{\prime }$ is a stress deviator. $E_{m}^{tan}$ is a tangent modulus obtained from a uniaxial tension or compression stress-strain curve. The corresponding tangent Poisson’s ratio and the tangent shear modulus are given by Equations (115) and (116).

(115) $$\;\upsilon_{m}^{tan}=\frac{1}{2}-\left(\frac{1}{2}-\upsilon_{m}\right)\frac{E_{m}^{tan}}{E_{m}}\;$$

(116) $$\;G_{m}^{tan}=\frac{E_{m}^{tan}}{2\left(1+\upsilon_{m}^{tan}\right)}\;$$

Owing to anisotropy of the matrix in an elasto-plastic region, the corresponding tangent Eshelby tensor has to be calculated by a numerical integration (see Equations (117)–(126)) [230].

(117) $$\;S_{ijkl}^{tan}=\frac{1}{8\pi }L_{mnkl}^{m-\tan}\overset{+1}{\underset{-1}{\int }}d\zeta_{3}\overset{2\pi }{\underset{0}{\int }}\left\{G_{imjn}\left(\bar{\xi }\right)+G_{j\min}\left(\bar{\xi }\right)\right\}d\omega \;$$

(118) $$\;L_{mnkl}^{m-tan}={\left[M_{mnkl}^{m-tan}\right]}^{-1}\;$$

(119) $$\;G_{ijkl}\left(\bar{\xi }\right)=\bar{\xi_{k}}\bar{\xi_{l}}N_{ij}(\bar{\xi })/D(\bar{\xi })\;$$

(120) $$\;N_{ij}\left(\bar{\xi }\right)=\frac{1}{2}\varepsilon_{ikl}\varepsilon_{jmn}K_{km}K_{ln}\;$$

(121) $$\;D\left(\bar{\xi }\right)=\varepsilon_{mnl}K_{m1}K_{n2}K_{l3}\;$$

(122) $$\;K_{ik}=M_{ijkl}^{m-tan}\bar{\xi_{j}}\bar{\xi_{l}}\;$$

(123) $$\;\varepsilon_{ijk}=\frac{1}{2}\left(i-j\right)\left(j-k\right)\left(k-i\right)\;$$

(124) $$\;\bar{\xi }=\zeta_{i}/a_{i}\;$$

(125) $$\;\zeta_{1}={\left(1-\zeta_{3}^{2}\right)}^{1/2}\cos\omega \;$$

(126) $$\;\zeta_{2}={\left(1-\zeta_{3}^{2}\right)}^{1/2}\sin\omega \;$$

The superscript “*m-tan*” denotes quantities of the matrix in the tangent form.

#### 4.3.3. Transformation Field Analysis (TFA)

Dvorak [208,281] proposed a transformation field analysis to evaluate elastoplastic behaviors of a composite, in which the plastic strain of a matrix is viewed as an eigenstrain. The stress-strain relations of the composite and the constituent phases are presented in Equations (127)–(130).

(127) $$\;d\sigma =L\left(d\varepsilon -d\varepsilon^{pla}\right)=Ld\varepsilon +d\lambda \;$$

(128) $$\;d\varepsilon =Md\sigma +d\varepsilon^{pla}\;$$

(129) $$\;d\sigma_{r}=L_{r}\left(d\varepsilon_{r}-d\varepsilon_{r}^{pla}\right)=L_{r}d\varepsilon_{r}+d\lambda_{r},\;r=f,m\;$$

(130) $$\;d\varepsilon_{r}=M_{r}d\sigma_{r}+d\varepsilon_{r}^{pla},\;r=f,m\;$$

where dλ, $d\lambda_{r}$, and $d\varepsilon^{pla}$, $d\varepsilon_{r}^{pla}$ denote eigenstresses and eigenstrains of the composite and the constituents. Obviously, $d\lambda =-Ld\varepsilon^{pla}$ and $d\lambda_{r}=-Ld\varepsilon_{r}^{pla}$. $L_{r}$ and $M_{r}$ are the elastic stiffness and compliance tensors of the constituents, with L and M being the elastic stiffness and compliance tensors of the composite. The localization rule is shown as Equations (131)–(134).

(131) $$\;d\varepsilon_{r}=A_{r}d\varepsilon +\underset{s}{\sum }D_{rs}d\varepsilon_{s}^{pla},\;r,s=f,m\;$$

(132) $$\;d\sigma_{r}=B_{r}d\sigma +\underset{s}{\sum }F_{rs}d\lambda_{s},\;r,s=f,m\;$$

(133) $$\;d\varepsilon^{pla}=\underset{r}{\sum }V_{r}B_{r}^{T}d\varepsilon_{r}^{pla},\;r=f,m\;$$

(134) $$\;d\lambda =\underset{r}{\sum }V_{r}A_{r}^{T}d\lambda_{r},\;r=f,m\;$$

where $A_{r}$ and $B_{r}$ are the global strain and stress concentration tensors, respectively. $A_{r}^{T}$ and $B_{r}^{T}$ are corresponding transposed tensors. $D_{rs}$ and $F_{rs}$ are the influence tensors given by Equations (135)–(138),

(135) $$\;D_{rm}=\left(I-A_{r}\right){\left(L_{m}-L_{f}\right)}^{-1}L_{m},\;r=f,m\;$$

(136) $$\;D_{rf}=-\left(I-A_{r}\right){\left(L_{m}-L_{f}\right)}^{-1}L_{f},\;r=f,m\;$$

(137) $$\;F_{rm}=\left(I-B_{r}\right){\left(M_{m}-M_{f}\right)}^{-1}M_{m},\;r=f,m\;$$

(138) $$\;F_{rf}=-\left(I-B_{r}\right){\left(M_{m}-M_{f}\right)}^{-1}M_{f},\;r=f,m\;$$

It is reported by Chaboche [57,95] that the prediction results of elastoplastic behavior of composites provided by the traditional TFA are too stiff. Modifications to Equations (131)–(132) were proposed with the correction tensors $K_{r},\;r=f,m$, as shown in Equations (139) and (140) [57,95].

(139) $$\;d\varepsilon_{r}=A_{r}d\varepsilon +\underset{s}{\sum }D_{rs}K_{s}d\varepsilon_{s}^{pla},\;r,s=f,m\;$$

(140) $$\;d\sigma_{r}=B_{r}d\sigma -\underset{s}{\sum }F_{rs}L_{s}K_{s}d\varepsilon_{s}^{pla},\;r,s=f,m\;$$

The correction tensors can be obtained by solving Equation (141).

(141) $$\;\underset{s}{\sum }D_{rs}K_{s}\left(M_{s}^{tan}-M_{s}\right)L_{s}^{tan}A_{s}^{tan}=A_{r}^{tan}-A_{r},\;r,s=f,m\;$$

where $M_{s}^{tan},\;L_{s}^{tan}$ are the instantaneous tangent compliance and stiffness tensors of the constituents. Since the fiber is linearly elastic, only $M_{m}^{tan}$ needs to be updated. $A_{r}^{tan}$ are the instantaneous strain concentration tensors calculated from $M_{s}^{tan},\;L_{s}^{tan}$, and the related Eshelby tensor. As indicated by Chaboche [57], Equation (141) is indeterminate. But it can be solved by choosing $K_{s}=I$ for the stiffer constituent phase. For a two phase composite with the elastic fibers as reinforcement, we can get Equations (142) and (143).

(142) $$\;K_{f}=I\;$$

(143) $$\;K_{m}=\left(I-M_{m}L_{f}\right)\left(I-A_{m}^{-1}\right)\left({\left(A_{m}^{tan}\right)}^{-1}-{\left(A_{m}^{\;}\right)}^{-1}\right){\left(I-M_{m}L_{m}^{tan}\right)}^{-1}\;$$

#### 4.3.4. Affine Formulation

Zaoui and Masson [219] and Masson et al. [218] proposed a new linearization, namely an affine formulation, to improve the prediction accuracy of the viscoplastic behavior of a composite. Chaboche [95] presented a compact version of the affine formulation for an elasto-plastic case. Equations (144)–(147) are the corresponding formulations.

(144) $$\;\sigma =L^{tan}\varepsilon +\tau \;$$

(145) $$\;\varepsilon =M^{tan}\sigma +\eta \;$$

(146) $$\;\sigma_{r}=L_{r}^{tan}\varepsilon_{r}+\tau_{r},\;r=f,m\;$$

(147) $$\;\varepsilon_{r}=M_{r}^{tan}\sigma_{r}+\eta_{r},\;r=f,m\;$$

where τ, $\tau_{r}$ and η, $\eta_{r}$ are the pre-stresses and pre-strains of the composite and its constituents. The tangent instantaneous compliance tensor $M_{m}^{tan}$ is given in Equation (112), and $L_{m}^{tan}={\left(M_{m}^{tan}\right)}^{-1}$. The instantaneous effective compliance and stiffness tensors $M^{tan}$ and $L^{tan}$ are obtained from a selected homogenization model. The localization equations are given by Equations (148)–(150).

(148) $$\;\varepsilon_{r}=A_{r}^{tan}\varepsilon +A_{r}^{tan}S^{tan}M_{m}^{tan}\left(\tau -\tau_{r}\right)\;$$

(149) $$\;\tau =\underset{r}{\sum }V_{r}{\left(A_{r}^{tan}\right)}^{T}\tau_{r},\;r=f,m\;$$

(150) $$\;\eta =\underset{r}{\sum }V_{r}{\left(B_{r}^{tan}\right)}^{T}\eta_{r},\;r=f,m\;$$

In Equation (148), $S^{tan}$ is given by Equation (117). The instantaneous concentration tensors $A_{r}^{tan}$ and $B_{r}^{tan}$ can be calculated from a selected homogenization model, e.g., Mori–Tanaka model.

#### 4.3.5. Incremental-Secant Scheme

**Figure 19:**
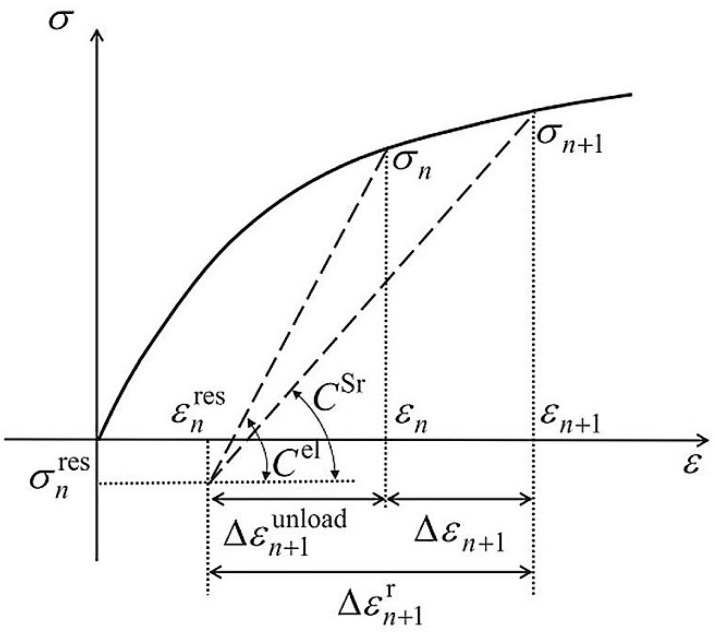
Schematic of the incremental-secant linearization [112].

Wu et al. [112] proposed a novel incremental-secant linearization for elasto-plastic responses of a composite. It is claimed that the linearization is applicable to non-monotonic and non-proportional loads [112,113,114]. Figure 19 illustrates the idea of the linearization.

Consider a composite at the *nth* load step with a total stress and strain tensors, $\sigma_{n}$ and $\varepsilon_{n}$. The corresponding stress and strain increments are $\Delta \sigma_{n+1},\;\Delta \varepsilon_{n+1}$. Then the total stress and strain tensors in the next step are given by Equations (151) and (152).

(151) $$\;\varepsilon_{n+1}=\varepsilon_{n}+\Delta \varepsilon_{n+1}\;$$

(152) $$\;\sigma_{n+1}=\sigma_{n}+\Delta \sigma_{n+1}\;$$

The subscripts *n* and (*n* + 1) denote quantities of the *n*th and (*n* + 1)th load steps, respectively. In the incremental-secant scheme, a fictitious elastic unload process is introduced at the *n*th load step. It should be noted that the residual stresses in constituent phases can be nonzero due to the heterogeneity even no homogenized residual stress exists in the composite. From a fictitious unload state $\left(\sigma_{n}^{res},\varepsilon_{n}^{res}\right)$, a secant reload procedure is applied from the *n*th to the (*n* + 1)th step. Thus, Equations (153)–(155) are obtained.

(153) $$\;\varepsilon_{n+1}=\varepsilon_{n}^{res}+\Delta \varepsilon_{n+1}^{rel}\;$$

(154) $$\;\sigma_{n+1}=\sigma_{n}^{res}+\Delta \sigma_{n+1}^{rel}\;$$

(155) $$\;\Delta \sigma_{n+1}^{rel}=L_{n+1}^{sec}\Delta \varepsilon_{n+1}^{rel}\;$$

$\varepsilon_{n}^{res}$ and $\sigma_{n}^{res}$ are the residual strains and stresses after unload. $L_{n+1}^{sec}$ is the secant stiffness tensor at the (*n* + 1)th load step, viewing $\left(\sigma_{n}^{res},\varepsilon_{n}^{res}\right)$ as a starting point. $\Delta \varepsilon_{n+1}^{rel}$ and $\Delta \sigma_{n+1}^{rel}$ are the reload strain and stress increments. It is assumed that at the *n*th step, all the quantities required are known. Given a total strain $\varepsilon_{n+1}$ for the next load step, the strain increment $\Delta \varepsilon_{n+1}^{rel}$ can be obtained from Equation (153). Consequently, the reload stress increment $\Delta \sigma_{n+1}^{rel}$ is reached by Equation (155), and the total stress $\sigma_{n+1}$ is obtained from Equation (154). It should be noted that the homogenized residual stress of the composite is zero, namely $\sigma_{n}^{res}=0$. Thus, in Equation (155), the only unknown argument is the effective secant stiffness tensor $L_{n+1}^{sec}$. It can be obtained from the secant properties of the constituents through Equations (156)–(157).

(156) $$\;\sigma_{n+1}^{i}-\sigma_{n}^{i-res}=L_{n+1}^{i-sec}\Delta \varepsilon_{n+1}^{i-rel},\;i=f,m\;$$

(157) $$\;\Delta \varepsilon_{n+1}^{i-rel}=A_{n+1}^{i-sec}\Delta \varepsilon_{n+1}^{rel},\;i=f,m\;$$

The secant stiffness tensor $L_{n+1}^{i-sec}$ and the secant global strain concentration tensor $A_{n+1}^{i-sec}$ depend on the stresses of the phases $\sigma_{n+1}^{i}$. Thus, $L_{n+1}^{i-sec}$, $A_{n+1}^{i-sec}$, and $\sigma_{n+1}^{i}$ can be obtained by solving Equations (156) and (157) iteratively. Then the homogenized stresses of the composite $\sigma_{n+1}$ can be determined from Equations (154) and (155), in which the $L_{n+1}^{sec}$ is evaluated from $L_{n+1}^{i-sec}$ and $A_{n+1}^{i-sec}$ by a selected homogenization theory. Alternatively, $\sigma_{n+1}^{\;}$ can also be obtained from Equation (158).

(158) $$\;\sigma_{n+1}^{\;}=V_{f}\sigma_{n+1}^{f}+V_{m}\sigma_{n+1}^{m}\;$$

To continue the calculation to next load step, it is necessary to get the residual stresses and strains of the composite and the constituents. The residual stresses of the composite are zero, and the corresponding residual strains are given by Equations (159) and (160):

(159) $$\;\varepsilon_{n+1}^{res}=\varepsilon_{n+1}^{\;}-\Delta \varepsilon_{n+1}^{unl}\;$$

(160) $$\;\Delta \varepsilon_{n+1}^{unl}=M^{ela}\sigma_{n+1}^{\;}\;$$

where $\Delta \varepsilon_{n+1}^{unl}$ are the unload strains shown in Figure 19. $M^{ela}$ is the elastic effective compliance tensor of the composite evaluated by a selected homogenization theory. The residual strains and stresses of the constituents are given by Equations (161) and (162).

(161) $$\;\varepsilon_{n+1}^{i-res}=\varepsilon_{n+1}^{i}-\Delta \varepsilon_{n+1}^{i-unl}\;$$

(162) $$\;\sigma_{n+1}^{i-res}=\sigma_{n+1}^{i}-L_{i}^{ela}\Delta \varepsilon_{n+1}^{i-unl}\;$$

The unload strains of the constituents are found as Equation (163).

(163) $$\;\Delta \varepsilon_{n+1}^{i-unl}=A_{n+1}^{i-ela}\Delta \varepsilon_{n+1}^{unl},\;i=f,m\;$$

In this way, the calculation process continues.

#### 4.3.6. Quantitative Comparison on the Linearizations

Before a quantitative study, the applicability of the five linearization models should be briefly introduced. Applicability of a linearization model can be assessed in three aspects, i.e., whether they are applicable to non-monotonic and non-proportional loads, whether a tension-shear coupling can be considered, and whether a numerical integration on an Eshelby tensor can be avoided. Table 9 summarizes the applicability of different linearization models. Essentially, if a linearization is in an incremental form, it is applicable to non-monotonic and non-proportional loading cases. Rewriting an elastoplastic compliance tensor into four blocks, a non-zero off-diagonal block implies that the model can account for a tension-shearing coupling. The third aspect depends on whether Equation (117) is used.

**Figure 20:**
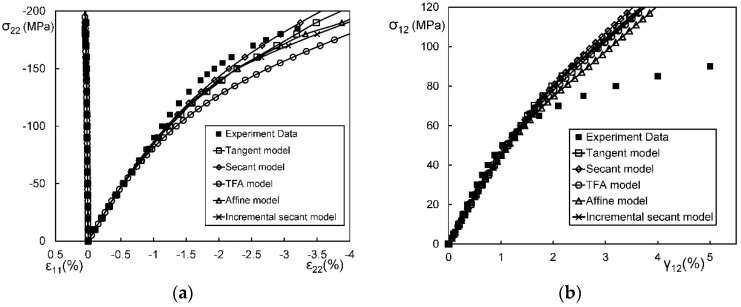
Comparison among linearizations–IM7/8551-7 UD composite. (**a**) Transverse compression; (**b**) In-plane shear.

**Figure 21:**
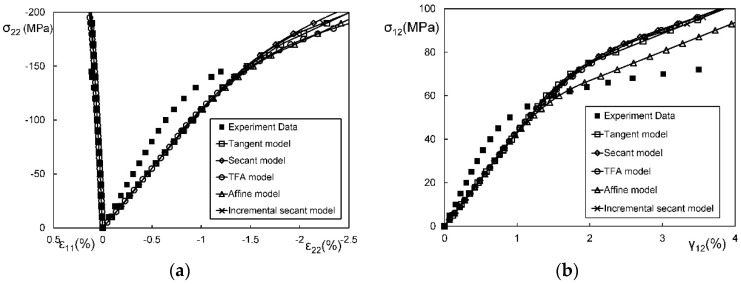
Comparison among linearizations–E-Glass/Epoxy UD composite. (**a**) Transverse compression; (**b**) In plane shear.

**Figure 22:**
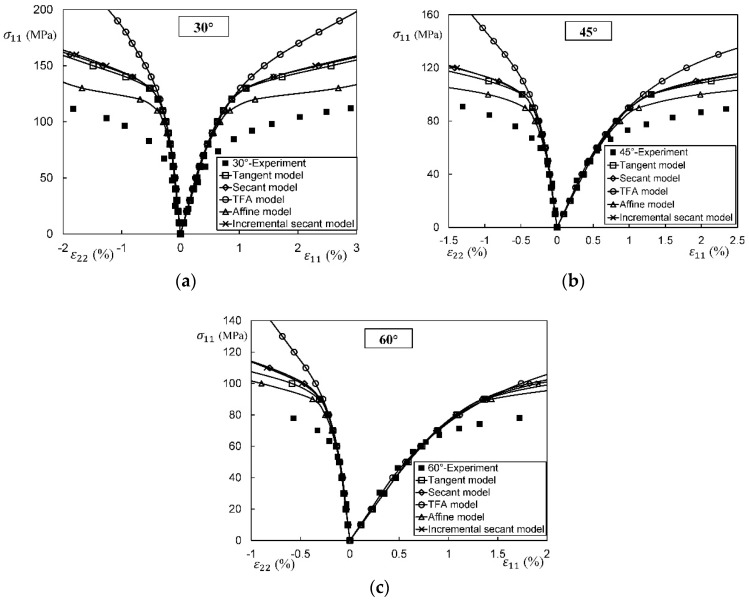
Comparison among linearizations–AS4/Peek UD composite. (**a**) 30° off-axis tension; (**b**) 45° off-axis tension; (**c**) 60° off-axis tension.

A quantitative comparison is made taken experimental results of E-glass/Epoxy, IM7/8551-7, and AS4/Peek UD composites as benchmark. The matrix behavior in the secant and the incremental-secant linearizations is taken as isotropic, whereas that in the other four linearizations can be either isotropic [212] or anisotropic. For a consistency in the comparison, the matrix is assumed to be elasto-plastically isotropic. Each linearization is incorporated with Mori–Tanaka model. Results are shown in Figure 20, Figure 21 and Figure 22. Again, the comparison results are restricted to static, monotonic, and proportional load cases.

Since all the five linearizations are combined with Mori–Tanaka model, errors induced by the predicted elastic moduli and yield points cannot be reflected. As for the predicted asymptotic tangent modulus, $E_{asy}^{T}$ or $G_{asy}^{T}$, it is seen that the affine formulation delivers a relative soft response, whereas the TFA’s predictions are stiffer for the cases in Figure 22a,b. The other four theories present similar results. Overall, the results from all of the five theories are stiff compared with the experiments, especially for the in-plane shear cases.

### 4.4. Comparison on Modifications of a Plastic Eshelby Tensor

Generally speaking, the Eshelby tensor for an anisotropic material should be obtained from a numerical integration. However, some works reported that the precise Eshelby tensor would overestimate the instantaneous stiffness of a composite [95,210,211,212]. Besides, the numerical integration is time consuming compared with an explicit one. Thus, several modifications on a plastic Eshelby tensor are proposed, e.g., an isotropic matrix approach, an isotropic Eshelby tensor approach, and Peng’s approach. Including the numerical integration, which is called an anisotropic Eshelby tensor approach, the four methods are compared.

In this section, all the modifications on Eshelby tensor are incorporated with Mori–Tanaka model, first-moment equivalent stress, and tangent linearization.

#### 4.4.1. Anisotropic Eshelby Tensor Approach

In this approach, an elastoplastic compliance or stiffness tensor of the matrix is evaluated by the J2 flow rule shown in Equations (112)–(114). The corresponding Eshelby tensor is obtained from Equation (117), and the effective properties of the composite are derived from Equation (109).

#### 4.4.2. Isotropic Matrix Method

González and LLorca [212] demonstrated that an elastoplastic tangent stiffness tensor of the matrix can be expressed as isotropic if the composite is subjected to an asymmetrically proportional load. In such a case, $M_{m}^{tan}$ and $S_{\;}^{tan}$ can be directly obtained by replacing the secant moduli $E_{m}^{sec}$, $G_{m}^{sec}$, and $\upsilon_{m}^{sec}$ in Equations (98)–(105) with the tangent ones, $E_{m}^{tan}$, $G_{m}^{tan}$, and $\upsilon_{m}^{tan}$.

#### 4.4.3. Isotropic Eshelby Tensor Method

It was suggested by Doghri and Ouaar [65] that a better evaluation of elastoplastic behavior of a composite could be achieved if the elasto-plastic response of the matrix remain anisotropic, but the corresponding Eshelby tensor is defined through an isotropic manner. Specifically, the expressions of $M_{m}^{tan}$ are the same by Equations (112)–(114). The Eshelby tensor is obtained by replacing $E_{m}^{sec}$, $G_{m}^{sec}$, and $\upsilon_{m}^{sec}$ in Equations (103)–(105) with $E_{m}^{tan}$, $G_{m}^{tan}$, and $\upsilon_{m}^{tan}$.

#### 4.4.4. Peng’s Approach

Peng et al. [116] presented a new method to determine an Eshelby tensor for the elastoplastic behavior of a composite. In Peng’s approach, a reference elastic medium is introduced, whose configuration and properties are identical to the elastoplastic matrix in the composite. It is assumed that the elastoplastic behavior of the composite can be characterized by two kinds of eigenstrains. One is induced by the inhomogeneity, and the other by the plastic deformation of the matrix. Based on this, a new determination of the Eshelby tensor is given by Equation (164) [116].

(164) $$\;S^{*}={\left[M_{m}^{\;}L_{m}^{tan}\right]}^{-1}S_{m}{}^{\;}\left[M_{m}^{\;}L_{m}^{tan}\right]\;$$

where $M_{m}^{\;}$ is an elastic compliance tensor of the matrix, and $S_{m}$ is the corresponding elastic Eshelby tensor. $L_{m}^{tan}$ is an instantaneous tangent stiffness tensor of the matrix. If Mori–Tanaka model is employed, the effective compliance tensor of the composite can be obtained by replacing $S_{m}^{tan}$ in Equations (109)–(111) with $S^{*}$ shown in Equation (164).

#### 4.4.5. Quantitative Comparison on Modifications of the Eshelby Tensor

**Figure 23:**
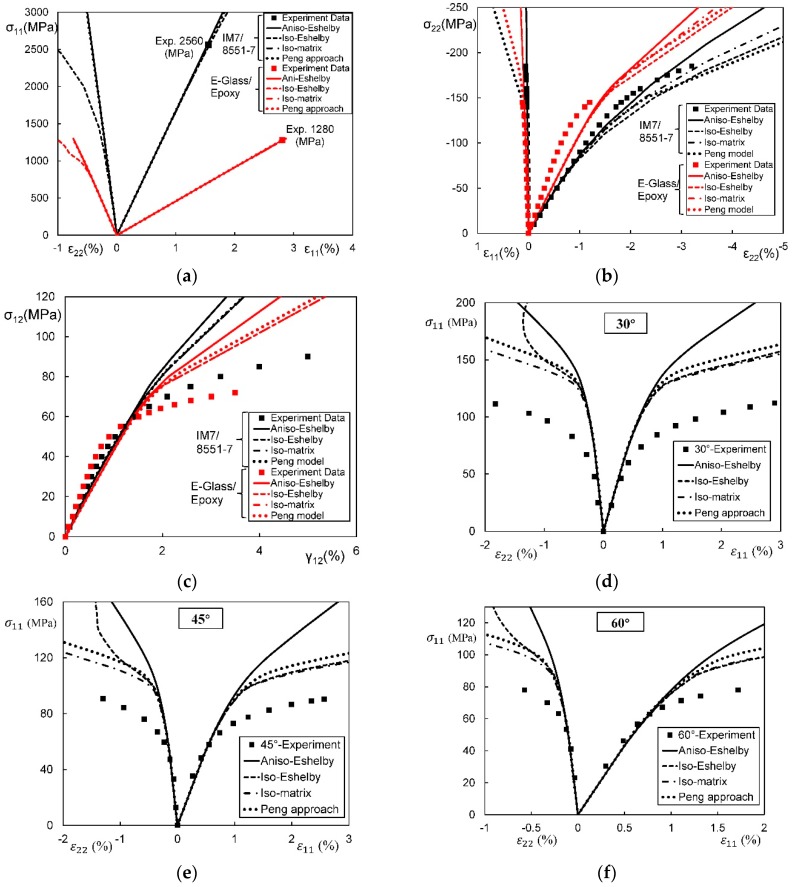
Comparison among the four approaches to determine an Eshelby tensor. (**a**) Longitudinal tension—(IM7/8551-7 UD composites); (**b**) Transverse compression—(IM7/8551-7 UD composites); (**c**) In-plane shear—(IM7/8551-7 UD composites); (**d**) 30° off-axis tension—(AS4/Peek UD composite); (**e**) 45° off-axis tension—(AS4/Peek UD composite); (**f**) 60° off-axis tension—(AS4/Peek UD composite).

Features of the four approaches to elasto-plastic behavior of a composite are summarized in Table 10. Amongst, the isotropic Eshelby tensor and Peng’s approaches can meet all the three aspects mentioned previously. Quantitative comparisons for the four approaches are shown in Figure 23.

From Figure 23a–f, we can see that the anisotropic Eshelby tensor approach presents much stiffer responses than the other three. However, the isotropic Eshelby tensor approach resulted in the $\sigma_{11}-\varepsilon_{22}$ curves in Figure 23a,d–f to be somewhat physically unacceptable. Table 11 lists the stresses of the constituent fiber and matrix predicted by the four approaches under some loads.

Table 11 indicates that the anisotropic Eshelby tensor, the isotropic matrix, and Peng’s approaches give similar predictions for the constituent stresses. However, the signs of the majority stress components predicted by the isotropic Eshelby tensor model are changed and the corresponding magnitudes are much larger than those by the three other approaches. Furthermore, a predicted tri-axial tension and tri-axial compression may be attained by the fiber and matrix, respectively, even though a composite is subjected to a longitudinal tension. Due to these, a further development in the isotropic Eshelby tensor model is expected.

Also, the $\sigma_{11}-\varepsilon_{22}$ curves predicted by Peng’s approach in Figure 23b looks quite different from the experiments. Besides, the tendency of the predicted curves by Peng’s approach in other cases was consistent with the experiments. Thus, Peng’s approach can be seen a reliable method to a large extent. But attention should be paid to the Peng’s approach when dealing with a Poisson’s effect of a composite under a transverse load.

As mentioned in Section 4, the prediction accuracy can be improved by making use of a second-moment estimation or SCFs of the matrix. Both may be efficient to decrease the too stiff response predicted by the anisotropic Eshelby tensor approach. Considering a reliable applicability, the anisotropic Eshelby tensor approach is still recommended over the three other approaches.

## 5. Conclusions

A review and comparative study is made for various elastoplastic micromechanics models of a composite. The comparative study has been carried out regarding four aspects. They are the selection of a homogenization, the modifications on yield stress, the selection of a linearization, and the determination of an Eshelby tensor. Some conclusions can be drawn as follows.

In an elastic range, bridging model performs the best. In an elastoplastic range, Mori–Tanaka model, SCM, bridging model, and Chamis model are often found applicable. Based on an isotropic matrix assumption and a tangent linearization, all of the four models deliver stiffer results for elastoplastic stress-strain curves of some composites. The deviation sources can be attributed to threefold, i.e., the elastic modulus, the yield point, and the asymptotic tangent modulus.

The deviation resulted from an overestimated yield stress can be reduced by using a second stress estimation or the SCFs of the matrix in the composite. The former is only useful when the stress field fluctuation in the composite is significant. The latter can be applied to a more general case. Further, a combination of the elastoplastic bridging model and the SCFs is recommended, owing to its applicability for non-monotonic and non-proportional loads, higher prediction accuracy, and computational efficiency with no evaluation of an Eshelby tensor. Nevertheless, both of the two modifications are less efficient for an overestimated asymptotic tangent modulus.

Different linearizations can give different evaluations on the asymptotic tangent modulus of an elasto-plastic response of the composite, but not significant in general. Thus, attention should be focused on when to apply a linearization. More applications and developments on the incremental-secant linearization are expected because it is efficient in computation, applicable for complicated loads, good in accuracy, and flexible in combination with various homogenizations.

Regarding four kinds of determinations for the Eshelby tensor in an elasto-plastic range, the anisotropic Eshelby tensor approach delivers much stiffer results. The other three approaches present better. But, the isotropic-matrix approach has only been demonstrated applicable to asymmetrically proportional loads. The remaining two approaches need further development on their prediction capability on a Poisson’s effect.

Unfortunately, most of the methods reviewed in this work cannot modify an overestimation on the asymptotic tangent modulus to a satisfactory extent, especially for in-plane shear cases. This might be because a perfect fiber/matrix interface bonding has been incorporated with all of the methods. Furthermore, the plastic behavior parameters of a matrix have been determined only from a uniaxial tensile stress-strain curve, defined by a so-called single-parameter plasticity theory. A two-parameter plasticity theory, i.e., both measured uniaxial tensile and in-plane shear stress-strain curves of the matrix are used to define its plastic behavior parameters, would be more pertinent. It is expected that better predictions for elasto-plastic responses of a composite can be achieved from development in both micromechanics models for the composite and plasticity theories for an isotropic matrix.

## Figures and Tables

**Table 1:** Qualitative comparison among models at different length scales.

Complexity	Macro-Scale Model	Meso-Scale Model	Micro-Scale Model
Complexity of plasticity	***	**	*
Complexity of computation	*	**	***
Complexity of experiments	***	**	*
Complexity of modeling	*	**	***

*** = high, ** = medium, * = low.

**Table 2:** Halpin–Tsai equations.

$\;\frac{P}{P_{m}}=\frac{1+\zeta \eta V_{f}}{1-\eta V_{f}},\;\eta =\frac{P_{f}/P_{m}-1}{P_{f}/P_{m}+\zeta }$			
P	$P_{f}$	$P_{m}$	ζ
$k_{22}$	$k^{f}$	$k^{m}$	$1-2\upsilon_{m}$
$G_{12}$	$G_{12}^{f}$	$G^{m}$	1
$G_{23}$	$G_{23}^{f}$	$G^{m}$	$1/\left(3-4\upsilon_{m}\right)$

**Table 3:** Averaged prediction error for *E*_22_ ($Error=\frac{1}{9}\overset{9}{\underset{i=1}{\sum }}abs{\left(error\right)}_{i}$).

Models	Average Error	Rank	Models	Average Error	Rank
Bridging Model	12.4%	1	GSCM	25.1%	7
SCM	14.3%	2	Halpin–Tsai Equations	28.2%	8
FVDAM	14.9%	3	Mori–Tanaka Model	28.2%	8
FEM	15.9%	4	Rule of Mixture	43.5%	10
GMC	18.4%	5	Eshelby model	44.3%	11
Chamis Model	21.4%	6	-	-	-

**Table 4:** Averaged prediction error for *G*_12_ ($Error=\frac{1}{9}\overset{9}{\underset{i=1}{\sum }}abs{\left(error\right)}_{i}$).

Models	Average Error	Rank	Models	Average Error	Rank
Bridging Model	14.6%	1	GSCM	25.2%	5
Chamis Model	18.1%	2	GMC	27.0%	8
FVDAM	22%	3	Rule of Mixture	48.1%	9
FEM	22.1%	4	Eshelby model	53.5%	10
Halpin–Tsai Equations	25.2%	5	SCM	62%	11
Mori–Tanaka Model	25.2%	5	-	-	-

**Table 5:** Averaged prediction error for *G*_23_ ($Error=\frac{1}{9}\overset{9}{\underset{i=1}{\sum }}abs{\left(error\right)}_{i}$).

Models	Average Error	Rank	Models	Average Error	Rank
FEM	8.8%	1	GSCM	22.4%	7
FVDAM	8.9%	2	Halpin–Tsai Equations	26.9%	8
Bridging Model	9%	3	Mori–Tanaka Model	26.9%	8
SCM	11.5%	4	Rule of Mixture	39.1%	10
GMC	11.8%	5	Eshelby model	45.5%	11
Chamis Model	15%	6	-	-	-

**Table 6:** Averaged prediction error for $\upsilon_{12}$ ($Error=\frac{1}{9}\overset{9}{\underset{i=1}{\sum }}abs{\left(error\right)}_{i}$).

Models	Average Error	Rank	Models	Average Error	Rank
Eshelby Model	7.3%	1	GSCM	14.9%	7
Rule of Mixture	12.9%	2	GMC	15%	8
Bridging Model	12.9%	2	FVDAM	15.3%	9
Chamis Model	12.9%	2	FEM	15.4%	10
Halpin–Tsai Equations	12.9%	2	SCM	18.3%	11
Mori–Tanaka Model	14.6%	6	-	-	-

**Table 7:** Overall averaged prediction error for the five constants $Error=\frac{1}{45}\overset{45}{\underset{i=1}{\sum }}abs{\left(error\right)}_{i}$.

Models	Average Error	Rank	Models	Average Error	Rank
Bridging model	10.38%	1	Halpin–Tsai equations	19.24%	7
FVDAM	12.83%	2	Mori–Tanaka model	19.59%	8
FEM	13.08%	3	SCM	21.82%	9
Chamis model	14.09%	4	Rule of mixture	28.4%	10
GMC	15.07%	5	Eshelby model	30.72%	11
GSCM	18.14%	6	-	-	-

**Table 8:** Averaged prediction errors of different models for the cases in Figure 9, Figure 10 and Figure 11.

Approaches	Tension-Shear Coupling	Error of E or G	Error of *σ^Y^*	Error of $E_{asy}^{T}\;$	$\;ER^{ov}\;$
Mori–Tanaka Model	Yes	18.5%	38.6%	134.8%	22.7%
Chamis model	No	14.2%	61.3%	154.8%	29.1%
Bridging model	Yes	12.6%	90.2%	206.1%	53.3%
Self-consistent model	Yes	28.0%	126.2%	845.8%	116.2%

**Table 9:** Comparison for the linearization methods.

Approaches	Non-Monotonic and Non-Proportional Load	Tension-Shear Coupling	Numerical Integration on ESHELBY TENSOR
Tangent model	Yes	Yes	Yes
Secant model	No	No	No
TFA model	Yes	No	No
Affine formulations	No	Yes	Yes
Incremental-secant scheme	Yes	No	No

**Table 10:** Applicability of different determinations of an Eshelby tensor.

Approaches	Non-Monotonic and Non-Proportional Load	Tension-Shear Coupling	Numerical Integration on Eshelby Tensor
Anisotropic Eshelby tensor	Yes	Yes	Yes
Isotropic matrix	No	No	No
Isotropic Eshelby tensor	Yes	Yes	No
Peng approach	Yes	Yes	No

**Table 11:** Stresses of constituent fiber and matrix under longitudinal tension.

Longitudinal Tension		$\;\sigma_{11}^{f}\;$	$\;\sigma_{22}^{f}\;\;$	$\;\sigma_{33}^{f}\;\;$	$\;\sigma_{11}^{m}\;$	$\;\sigma_{22}^{m}\;$	$\;\sigma_{33}^{m}\;$
IM7/8551-7 (2500 MPa)	Anisotropic Eshelby tensor	4132	−3	−3	51.7	4.6	4.6
	Isotropic Eshelby tensor	4303	191	191	−204	−286	−286
	Isotropic Matrix	4133	−2.4	−2.4	50.8	3.5	3.5
	Peng’s approach	4131	−3	−3	53.5	4.5	4.5
E-Glass/Epoxy (1300 MPa)	Anisotropic Eshelby tensor	2107	−4	−4	89	6	6
	Isotropic Eshelby tensor	2140	36	36	41	−53	−53
	Isotropic Matrix	2109	−3.4	−3.4	86	5.1	5.1
	Peng’s approach	2107	−4	−4	89	6	6

## Appendix A

The following tables show the detail information of the experimental data and simulation results of different models.

**Table A1:** Experiment data for nine kinds of UD composites system.

Composites		$\;E_{11}\;\left(GPa\right)\;$	$\;E_{22}\;\left(GPa\right)\;$	$\;G_{12}\;\left(GPa\right)\;$	$\;G_{23}\;\left(GPa\right)\;$	$\;\upsilon_{12}\;$
E-Glass *^a^*/LY556 (*V_f_* = 0.62)	Fiber	80	80	33.33	33.33	0.2
	Matrix	3.35	3.35	1.24	1.24	0.35
	Composite	53.5	17.7	5.83	6.32	0.28
E-Glass *^b^*/MY750 (*V_f_* = 0.60)	Fiber	74	74	30.8	30.8	0.2
	Matrix	3.35	3.35	1.24	1.24	0.35
	Composite	45.6	16.2	5.83	5.79	0.28
S2-Glass/Epoxy (*V_f_* = 0.60)	Fiber	87	87	36.3	36.3	0.2
	Matrix	3.2	3.2	1.19	1.19	0.35
	Composite	52	19	6.7	6.7	0.3
T300/BSL914C (*V_f_* = 0.60)	Fiber	230	15	15	7	0.2
	Matrix	4	4	1.48	1.48	0.35
	Composite	138	11	5.5	3.93	0.28
T300/PR319 (*V_f_* = 0.60)	Fiber	230	15	15	7	0.2
	Matrix	0.95	0.95	0.35	0.35	0.35
	Composite	129	5.6	1.33	1.86	0.32
AS carbon/Epoxy (*V_f_* = 0.60)	Fiber	231	15	15	7	0.2
	Matrix	3.2	3.2	1.19	1.19	0.35
	Composite	140	10	6	3.35	0.3
AS4/3501-6 (*V_f_* = 0.60)	Fiber	225	15	15	7	0.2
	Matrix	4.2	4.2	1.57	1.57	0.34
	Composite	126	11	6.6	3.93	0.28
IM7/8551-7 (*V_f_* = 0.60)	Fiber	276	19	27	7	0.2
	Matrix	4.08	4.08	1.48	1.48	0.38
	Composite	165	8.4	5.6	2.8	0.34
G40-800/5260 (*V_f_* = 0.60)	Fiber	290	19	27	7	0.2
	Matrix	3.45	3.45	1.28	1.28	0.35
	Composite	173	10	6.94	3.56	0.33

*^a^* E-Glass 21 × K43 Gevetex; *^b^* Silenka E-Glass 1200tex.

**Table A2:** Effective properties predicted by Eshelby model.

Composites	$\;E_{11}\;\left(GPa\right)\;$	$\;E_{22}\;\left(GPa\right)\;$	$\;G_{12}\;\left(GPa\right)\;$	$\;G_{23}\;\left(GPa\right)\;$	$\;\upsilon_{12}\;$
E-Glass *^a^*/LY556 (*V_f_* = 0.62)	50.8	7.15	2.67	2.42	0.28
E-Glass *^b^*/MY750 (*V_f_* = 0.60)	45.7	7.01	2.61	2.37	0.28
S2-Glass/Epoxy (*V_f_* = 0.60)	53.4	6.76	2.52	2.28	0.28
T300/BSL914C (*V_f_* = 0.60)	139.5	7.08	2.94	2.49	0.27
T300/PR319 (*V_f_* = 0.60)	138.4	1.98	0.75	0.67	0.28
AS carbon/Epoxy (*V_f_* = 0.60)	139.8	5.91	2.40	2.05	0.27
AS4/3501-6 (*V_f_* = 0.60)	136.6	7.30	3.09	2.60	0.27
IM7/8551-7 (*V_f_* = 0.60)	167.1	7.73	3.07	2.50	0.29
G40-800/5260 (*V_f_* = 0.60)	175.3	6.52	2.67	2.19	0.28
Average error	3.1%	44.3%	53.5%	45.5%	7.3%

*^a^* E-Glass 21 × K43 Gevetex; *^b^* Silenka E-Glass 1200tex.

**Table A3:** Effective properties predicted by SCM.

Composites	$\;E_{11}\;\left(GPa\right)\;$	$\;E_{22}\;\left(GPa\right)\;$	$\;G_{12}\;\left(GPa\right)\;$	$\;G_{23}\;\left(GPa\right)\;$	$\;\upsilon_{12}\;$
E-Glass *^a^*/LY556 (*V_f_* = 0.62)	50.9	18.91	11.34	6.96	0.23
E-Glass *^b^*/MY750 (*V_f_* = 0.60)	45.8	16.80	9.80	6.15	0.24
S2-Glass/Epoxy (*V_f_* = 0.60)	53.6	17.39	10.94	6.36	0.23
T300/BSL914C (*V_f_* = 0.60)	139.7	8.99	6.25	3.43	0.25
T300/PR319 (*V_f_* = 0.60)	138.4	4.19	4.18	1.55	0.24
AS carbon/Epoxy (*V_f_* = 0.60)	139.9	8.06	5.82	3.06	0.25
AS4/3501-6 (*V_f_* = 0.60)	136.7	9.14	6.37	3.53	0.25
IM7/8551-7 (*V_f_* = 0.60)	167.3	10.37	9.36	3.52	0.26
G40-800/5260 (*V_f_* = 0.60)	175.4	9.33	8.98	3.25	0.25
Average error	3.1%	14.3%	61.9%	11.5%	18.3%

*^a^* E-Glass 21 × K43 Gevetex; *^b^* Silenka E-Glass 1200tex.

**Table A4:** Effective properties predicted by Mori–Tanaka model.

Composites	$\;E_{11}\;\left(GPa\right)\;$	$\;E_{22}\;\left(GPa\right)\;$	$\;G_{12}\;\left(GPa\right)\;$	$\;G_{23}\;\left(GPa\right)\;$	$\;\upsilon_{12}\;$
E-Glass *^a^*/LY556 (*V_f_* = 0.62)	50.9	11.7	4.60	4.06	0.25
E-Glass *^b^*/MY750 (*V_f_* = 0.60)	45.8	11.02	4.32	3.83	0.25
S2-Glass/Epoxy (*V_f_* = 0.60)	53.5	10.78	4.23	3.72	0.25
T300/BSL914C (*V_f_* = 0.60)	139.6	8.57	4.35	3.21	0.26
T300/PR319 (*V_f_* = 0.60)	138.4	3.02	1.30	1.06	0.25
AS carbon/Epoxy (*V_f_* = 0.60)	139.9	7.48	3.67	2.77	0.26
AS4/3501-6 (*V_f_* = 0.60)	136.7	8.76	4.53	3.32	0.26
IM7/8551-7 (*V_f_* = 0.60)	167.3	9.67	4.92	3.23	0.27
G40-800/5260 (*V_f_* = 0.60)	175.4	8.47	4.36	2.92	0.25
Average error	3.1%	28.2%	25.2%	26.9%	14.6%

*^a^* E-Glass 21 × K43 Gevetex; *^b^* Silenka E-Glass 1200tex.

**Table A5:** Effective properties predicted by GSCM.

Composites	$\;E_{11}\;\left(GPa\right)\;$	$\;E_{22}\;\left(GPa\right)\;$	$\;G_{12}\;\left(GPa\right)\;$	$\;G_{23}\;\left(GPa\right)\;$	$\;\upsilon_{12}\;$
E-Glass *^a^*/LY556 (*V_f_* = 0.62)	50.9	12.87	4.6	4.65	0.25
E-Glass *^b^*/MY750 (*V_f_* = 0.60)	45.8	12.03	4.32	4.33	0.25
S2-Glass/Epoxy (*V_f_* = 0.60)	53.5	11.8	4.23	4.25	0.25
T300/BSL914C (*V_f_* = 0.60)	139.6	8.77	4.35	3.32	0.26
T300/PR319 (*V_f_* = 0.60)	138.4	3.27	1.29	1.19	0.25
AS carbon/Epoxy (*V_f_* = 0.60)	139.9	7.72	3.67	2.9	0.26
AS4/3501-6 (*V_f_* = 0.60)	136.7	8.93	4.54	3.42	0.25
IM7/8551-7 (*V_f_* = 0.60)	167.3	10.1	4.92	3.42	0.27
G40-800/5260 (*V_f_* = 0.60)	175.4	8.85	4.35	3.09	0.25
Average error	3.1%	25.1%	25.2%	22.4%	14.9%

*^a^* E-Glass 21 × K43 Gevetex; *^b^* Silenka E-Glass 1200tex.

**Table A6:** Effective properties predicted by rule of mixture.

Composites	$\;E_{11}\;\left(GPa\right)\;$	$\;E_{22}\;\left(GPa\right)\;$	$\;G_{12}\;\left(GPa\right)\;$	$\;G_{23}\;\left(GPa\right)\;$	$\;\upsilon_{12}\;$
E-Glass *^a^*/LY556 (*V_f_* = 0.62)	50.9	8.252	3.076	3.076	0.26
E-Glass *^b^*/MY750 (*V_f_* = 0.60)	45.7	7.84	2.92	2.92	0.26
S2-Glass/Epoxy (*V_f_* = 0.60)	53.5	7.58	2.82	2.82	0.26
T300/BSL914C (*V_f_* = 0.60)	139.6	7.14	3.225	2.811	0.26
T300/PR319 (*V_f_* = 0.60)	138.4	2.169	0.85	0.82	0.26
AS carbon/Epoxy (*V_f_* = 0.60)	139.9	6.1	2.65	2.36	0.26
AS4/3501-6 (*V_f_* = 0.60)	136.7	7.39	3.39	2.93	0.26
IM7/8551-7 (*V_f_* = 0.60)	167.2	7.72	3.42	2.81	0.27
G40-800/5260 (*V_f_* = 0.60)	175.4	6.78	2.99	2.51	0.26
Average error	3.1%	41.5%	48.1%	36.5%	12.9%

*^a^* E-Glass 21 × K43 Gevetex; *^b^* Silenka E-Glass 1200tex.

**Table A7:** Effective properties predicted by Chamis model.

Composites	$\;E_{11}\;\left(GPa\right)\;$	$\;E_{22}\;\left(GPa\right)\;$	$\;G_{12}\;\left(GPa\right)\;$	$\;G_{23}\;\left(GPa\right)\;$	$\;\upsilon_{12}\;$
E-Glass *^a^*/LY556 (*V_f_* = 0.62)	50.9	13.64	5.13	5.13	0.26
E-Glass *^b^*/MY750 (*V_f_* = 0.60)	45.7	12.86	4.83	4.83	0.26
S2-Glass/Epoxy (*V_f_* = 0.60)	53.5	12.60	4.73	4.73	0.26
T300/BSL914C (*V_f_* = 0.60)	139.6	9.26	4.91	3.81	0.26
T300/PR319 (*V_f_* = 0.60)	138.4	3.46	1.45	1.33	0.27
AS carbon/Epoxy (*V_f_* = 0.60)	139.9	8.19	4.14	3.33	0.26
AS4/3501-6 (*V_f_* = 0.60)	136.7	9.50	5.12	3.93	0.26
IM7/8551-7 (*V_f_* = 0.60)	167.2	10.42	5.52	3.80	0.27
G40-800/5260 (*V_f_* = 0.60)	175.4	9.43	4.88	3.49	0.26
Average error	3.1%	21.4%	18.1%	15.0%	12.9%

*^a^* E-Glass 21 × K43 Gevetex; *^b^* Silenka E-Glass 1200tex.

**Table A8:** Effective properties predicted by Halpin–Tsai equations.

Composites	$\;E_{11}\;\left(GPa\right)\;$	$\;E_{22}\;\left(GPa\right)\;$	$\;G_{12}\;\left(GPa\right)\;$	$\;G_{23}\;\left(GPa\right)\;$	$\;\upsilon_{12}\;$
E-Glass *^a^*/LY556 (*V_f_* = 0.62)	50.9	11.7	4.6	4.06	0.26
E-Glass *^b^*/MY750 (*V_f_* = 0.60)	45.7	11.02	4.32	3.83	0.26
S2-Glass/Epoxy (*V_f_* = 0.60)	53.5	10.78	4.23	3.72	0.26
T300/BSL914C (*V_f_* = 0.60)	139.6	8.57	4.35	3.21	0.26
T300/PR319 (*V_f_* = 0.60)	138.4	3.02	1.29	1.06	0.27
AS carbon/Epoxy (*V_f_* = 0.60)	139.9	7.48	3.67	2.77	0.26
AS4/3501-6 (*V_f_* = 0.60)	136.7	8.76	4.54	3.32	0.26
IM7/8551-7 (*V_f_* = 0.60)	167.2	9.67	4.92	3.23	0.27
G40-800/5260 (*V_f_* = 0.60)	175.4	8.47	4.35	2.92	0.26
Average error	3.1%	28.2%	25.2%	26.9%	12.9%

*^a^* E-Glass 21 × K43 Gevetex; *^b^* Silenka E-Glass 1200tex.

**Table A9:** Effective properties predicted by bridging model (α=β=0.3).

Composites	$\;E_{11}\;\left(GPa\right)\;$	$\;E_{22}\;\left(GPa\right)\;$	$\;G_{12}\;\left(GPa\right)\;$	$\;G_{23}\;\left(GPa\right)\;$	$\;\upsilon_{12}\;$
E-Glass *^a^*/LY556 (*V_f_* = 0.62)	50.9	18.1	6.28	6.24	0.26
E-Glass *^b^*/MY750 (*V_f_* = 0.60)	45.7	16.8	5.84	5.8	0.26
S2-Glass/Epoxy (*V_f_* = 0.60)	53.5	16.9	5.81	5.77	0.26
T300/BSL914C (*V_f_* = 0.60)	139.6	9.6	5.35	3.66	0.26
T300/PR319 (*V_f_* = 0.60)	138.4	4.41	1.82	1.55	0.27
AS carbon/Epoxy (*V_f_* = 0.60)	139.9	8.7	4.64	3.29	0.26
AS4/3501-6 (*V_f_* = 0.60)	136.7	9.7	5.54	3.76	0.26
IM7/8551-7 (*V_f_* = 0.60)	167.2	11.2	6.46	3.76	0.27
G40-800/5260 (*V_f_* = 0.60)	175.4	10.2	5.8	3.51	0.26
Average error	3.1%	12.4%	14.6%	9.0%	12.9%

*^a^* E-Glass 21 × K43 Gevetex; *^b^* Silenka E-Glass 1200tex.

**Table A10:** Effective properties predicted by FEM.

Composites	$\;E_{11}\;\left(GPa\right)\;$	$\;E_{22}\;\left(GPa\right)\;$	$\;G_{12}\;\left(GPa\right)\;$	$\;G_{23}\;\left(GPa\right)\;$	$\;\upsilon_{12}\;$
E-Glass *^a^*/LY556 (*V_f_* = 0.62)	50.9	16.26	4.96	6.49	0.25
E-Glass *^b^*/MY750 (*V_f_* = 0.60)	45.8	14.9	4.58	5.89	0.25
S2-Glass/Epoxy (*V_f_* = 0.60)	53.5	14.86	4.5	5.89	0.25
T300/BSL914C (*V_f_* = 0.60)	139.6	9.42	4.5	3.71	0.26
T300/PR319 (*V_f_* = 0.60)	138.4	3.98	1.38	1.58	0.25
AS carbon/Epoxy (*V_f_* = 0.60)	139.9	8.45	3.82	3.32	0.26
AS4/3501-6 (*V_f_* = 0.60)	136.7	9.54	4.68	3.79	0.25
IM7/8551-7 (*V_f_* = 0.60)	167.2	10.88	5.15	3.79	0.27
G40-800/5260 (*V_f_* = 0.60)	175.4	9.63	4.57	3.47	0.25
Average error	3.1%	15.9%	22.1%	8.8%	15.4%

*^a^* E-Glass 21 × K43 Gevetex; *^b^* Silenka E-Glass 1200tex.

**Table A11:** Effective properties predicted by finite volume direct averaging micromechanics (FVDAM).

Composites	$\;E_{11}\;\left(GPa\right)\;$	$\;E_{22}\;\left(GPa\right)\;$	$\;G_{12}\;\left(GPa\right)\;$	$\;G_{23}\;\left(GPa\right)\;$	$\;\upsilon_{12}\;$
E-Glass *^a^*/LY556 (*V_f_* =0.62)	50.9	16.26	4.95	6.49	0.25
E-Glass *^b^*/MY750 (*V_f_* =0.60)	45.8	14.90	4.57	5.89	0.25
S2-Glass/Epoxy (*V_f_* = 0.60)	53.5	14.86	4.50	5.89	0.25
T300/BSL914C (*V_f_* = 0.60)	139.6	9.42	4.51	3.71	0.26
T300/PR319 (*V_f_* = 0.60)	138.4	3.97	1.38	1.57	0.25
AS carbon/Epoxy (*V_f_* = 0.60)	139.9	8.44	3.83	3.32	0.25
AS4/3501-6 (*V_f_* = 0.60)	136.7	9.53	4.70	3.79	0.25
IM7/8551-7 (*V_f_* = 0.60)	167.2	10.87	5.18	3.78	0.27
G40-800/5260 (*V_f_* = 0.60)	175.4	9.62	4.60	3.47	0.25
Average error	3.1%	14.9%	22%	8.9%	15.3%

*^a^* E-Glass 21 × K43 Gevetex; *^b^* Silenka E-Glass 1200tex.

**Table A12:** Effective properties predicted by generalized method of cells (GMC).

Composites	$\;E_{11}\;\left(GPa\right)\;$	$\;E_{22}\;\left(GPa\right)\;$	$\;G_{12}\;\left(GPa\right)\;$	$\;G_{23}\;\left(GPa\right)\;$	$\;\upsilon_{12}\;$
E-Glass *^a^*/LY556 (*V_f_* = 0.62)	50.9	15.40	4.57	6.02	0.25
E-Glass *^b^*/MY750 (*V_f_* = 0.60)	45.8	14.16	4.21	5.49	0.25
S2-Glass/Epoxy (*V_f_* = 0.60)	53.5	14.09	4.13	5.47	0.25
T300/BSL914C (*V_f_* = 0.60)	139.6	9.20	4.21	3.58	0.26
T300/PR319 (*V_f_* = 0.60)	138.4	3.78	1.27	1.47	0.25
AS carbon/Epoxy (*V_f_* = 0.60)	139.9	8.21	3.56	3.19	0.26
AS4/3501-6 (*V_f_* = 0.60)	136.7	9.33	4.39	3.67	0.25
IM7/8551-7 (*V_f_* = 0.60)	167.2	10.57	4.78	3.65	0.27
G40-800/5260 (*V_f_* = 0.60)	175.4	9.35	4.24	3.35	0.25
Average error	3.1%	18.4%	27.0%	11.8%	15.0%

*^a^* E-Glass 21 × K43 Gevetex; *^b^* Silenka E-Glass 1200tex.
